# medna-metadata: an open-source data management system for tracking environmental DNA samples and metadata

**DOI:** 10.1093/bioinformatics/btac556

**Published:** 2022-08-12

**Authors:** M Kimble, S Allers, K Campbell, C Chen, L M Jackson, B L King, S Silverbrand, G York, K Beard

**Affiliations:** School of Computing and Information Science, University of Maine, Orono, ME 04469, USA; Department of Molecular and Biomedical Sciences, University of Maine, Orono, ME 04469, USA; School of Computing and Information Science, University of Maine, Orono, ME 04469, USA; School of Computing and Information Science, University of Maine, Orono, ME 04469, USA; Advanced Research Computing, Security and Information Management, University of Maine, Orono, ME 04469, USA; Maine EPSCoR, University of Maine, Orono, ME 04469, USA; Department of Molecular and Biomedical Sciences, University of Maine, Orono, ME 04469, USA; School of Marine Sciences, University of Maine, Orono, ME 04469, USA; Environmental DNA Laboratory, Coordinated Operating Research Entities, University of Maine, Orono, ME 04469, USA; School of Computing and Information Science, University of Maine, Orono, ME 04469, USA

## Abstract

**Motivation:**

Environmental DNA (eDNA), as a rapidly expanding research field, stands to benefit from shared resources including sampling protocols, study designs, discovered sequences, and taxonomic assignments to sequences. High-quality community shareable eDNA resources rely heavily on comprehensive metadata documentation that captures the complex workflows covering field sampling, molecular biology lab work, and bioinformatic analyses. There are limited sources that provide documentation of database development on comprehensive metadata for eDNA and these workflows and no open-source software.

**Results:**

We present medna-metadata, an open-source, modular system that aligns with Findable, Accessible, Interoperable, and Reusable guiding principles that support scholarly data reuse and the database and application development of a standardized metadata collection structure that encapsulates critical aspects of field data collection, wet lab processing, and bioinformatic analysis. Medna-metadata is showcased with metabarcoding data from the Gulf of Maine (Polinski *et al.*, 2019).

**Availability and implementation:**

The source code of the medna-metadata web application is hosted on GitHub (https://github.com/Maine-eDNA/medna-metadata). Medna-metadata is a docker-compose installable package. Documentation can be found at https://medna-metadata.readthedocs.io/en/latest/?badge=latest. The application is implemented in Python, PostgreSQL and PostGIS, RabbitMQ, and NGINX, with all major browsers supported. A demo can be found at https://demo.metadata.maine-edna.org/.

**Supplementary information:**

Supplementary data are available at *Bioinformatics* online.

## 1 Introduction

Maine-eDNA is a state-wide research effort to determine the efficacy of ecosystem monitoring through environmental DNA (eDNA, https://umaine.edu/edna/). eDNA is DNA extracted from materials collected from the environment (e.g. air, water, sediment), and is an application domain that is increasingly applied to study the ecology of aquatic and other environments (Harrison *et al.*, 2019). Rapid growth has, however, resulted in growing pains stemming from poor data documentation practices (Nicholson *et al.*, 2020). Maine-eDNA endeavors to increase the ecological knowledge and sustainable use of Maine’s coastal ecosystems through multi-institutional collaboration. Maine-eDNA has hundreds of collaborators simultaneously collecting material samples in the field, extracting DNA from these samples, generating sequences through metabarcoding and using bioinformatic pipelines for quality filtering and taxonomic annotation of those sequences. At the onset of the program, a critical requirement was to have user-based access to results (annotated sequences) and metadata. This requirement led to the modeling and development of a geospatial-enabled database web application to support research efforts within the grant.

A general goal, and requirement for federal funding and publication, is to share and preserve genetic sequences by submission to an open access database such as GenBank and the Short Read Archive (National Science Foundation Biological Sciences Guidance on Data Management Plans, https://www.nsf.gov/bio/pubs/BIODMP_Guidance.pdf; Wilkinson *et al.*, 2016). To submit to these databases, data must adhere to minimum required fields and structure prior to submission. Some community standards pertinent to eDNA include: The Minimum Information about a Genome Sequence (MIGS, Field *et al.*, 2008) and Minimum Information for the Publication of Quantitative Real-Time PCR Experiments (MIQE, Bustin *et al.*, 2009). These minimum standards have branched into domain-specific checklists, such as minimum information about any (x) sequence (MIxS, Yilmaz *et al.*, 2011). Metabarcoding results are sensitive to each step in their generation (Bruce *et al.*, 2021), and while MIGS and MIQE standards provide guidance and structure, they are, as the name implies—a minimum. To increase confidence that detected change of an ecological state is due to actual change, rather than data artifacts, we found the need to supplement these standards with additional metadata that addresses sources of potential variation (Mathieu *et al.*, 2020).

From an informal survey of data collection habits of researchers project-wide, we found that recorded information was typically unstructured. In the absence of structured data collection protocols and a controlled vocabulary, we observed variation in terminology and naming, including variation in the naming of the same collection sites over time. There was also a routine practice of repeat manual modification of personalized documentation, which can increase the potential for epistemic and linguistic sources of uncertainty (Regan *et al.*, 2002). Most importantly, these idiosyncrasies can lead to incomparable datasets and limitations to reproducibility (Andersson *et al.*, 2020).

To maintain data integrity, enable meta-analyses, and provide efficient submission to open access databases, we needed to establish better control over documentation procedures. Thus spawned our efforts to develop a standardized data collection structure and vocabulary to encapsulate critical aspects of field data collection to wet lab processing to taxonomic annotation.

As noted by Wilkinson *et al.* (2016), publishing biodiversity data is largely a process of making species occurrence data findable, accessible, interoperable, and reusable (FAIR). FAIR principles suggest that all included metadata should have a globally unique and persistent identifier, thus making it ‘findable’ in a standardized and ‘accessible’ format. Haendel *et al.* (2016) suggests that when making data accessible, direct database endpoints are more valuable when integrated with a well-structured and provisioned application programming interface (API). In addition to accessibility principles, data should adopt a formal, broadly acceptable language for knowledge representation in an ‘interoperable’ manner and include well documented data versioning and vocabularies that follow FAIR principles. Data should be richly described with a plurality of accurate and relevant attributes, meet domain-relevant community standards, and include detailed provenance information making it ‘reusable’ (Wilkinson *et al.*, 2016). Beyond the core principles of FAIR data, it is also important that mechanisms exist to apply these principles equally, making the data both human and machine-readable (Rodríguez-Iglesias *et al.*, 2016). For eDNA data, reuse can be achieved by addressing spatial, temporal, laboratory, taxonomic, and other reporting inconsistencies and processes of standardization (Andersson *et al.*, 2020).

**Fig. 1.**
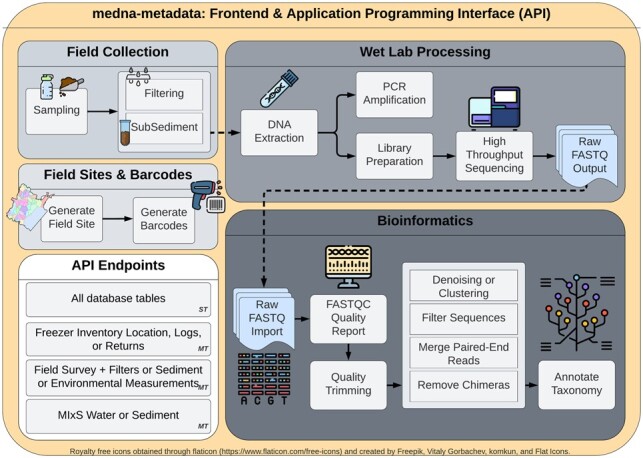
medna-metadata backend and frontend workflow spanning field sites, barcodes (sample labels), field collection, wet lab processing and bioinformatics. Single table (ST) application programming interface (API) endpoints are available for all tables within medna-metadata. Included are multi-table (MT), or custom API endpoints that provide multi-table join summaries

In a review of the literature and open-source repositories, we did not find any detailed database development covering comprehensive metadata from field data collection through wet lab processing to bioinformatics. Developing end-to-end database coverage was vital for the success of our project, and important for making sure our database adopted the FAIR guiding principles that support scholarly data reuse. We endeavored to provide a free and open-source database web application and API to increase the transparency of metadata operations (Fig. 1).

**Fig. 2.**
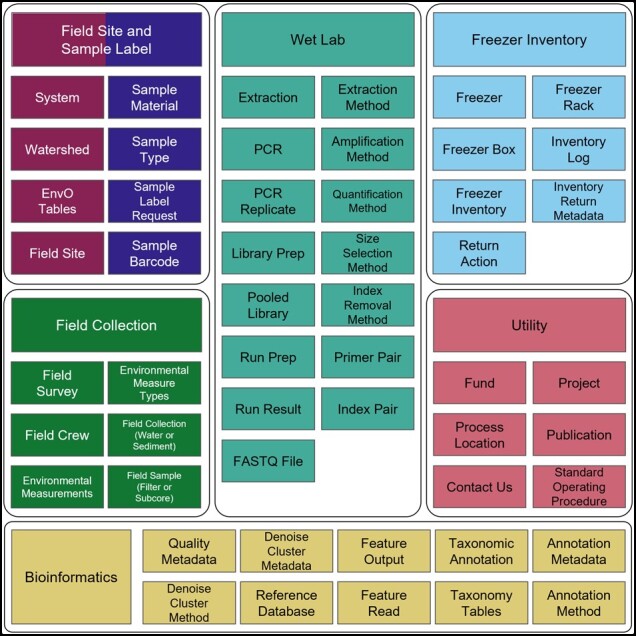
Primary tables and associated modules (utility, field site, sample label, field collection/survey, wet lab, freezer inventory, and bioinformatics) for medna-metadata. Full schema spanning the seven modules is available in Supplementary Figure S1. Full description of fields within tables is available in Supplementary Tables S1–S7

The remainder of this paper describes our steps, rationale, and components toward building reusable data through comprehensive metadata documentation. We present the details of our database development through an entity relationship diagram (ERD; full schema in Supplementary Fig. S1) which documents the schema for field data collection, wet lab processing, freezer inventory tracking, and bioinformatics (Fig. 2).

## 2 System and methods

### 2.1 Database modeling and design

#### 2.1.1 Field sites and sample labels

**Fig. 3.**
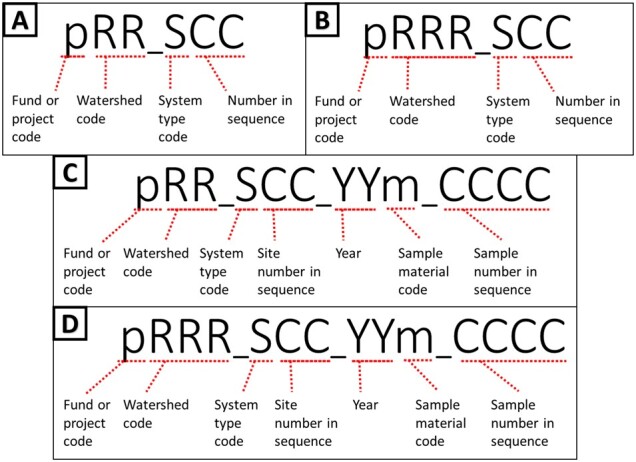
Site ID naming conventions: pRR_SCC where p is the fund, or project code, RR (**A**, **C**) or RRR (B, D) is the watershed region code, S is the system type, and CC is the two-digit sequence. Naming conventions for sample barcode labels (**C**, **D**): pRR_SCC_YYm_CCCC where pRR_SCC is the Site ID, m is the sample material code, and CCCC is the four-digit sequence

**Fig. 4.**
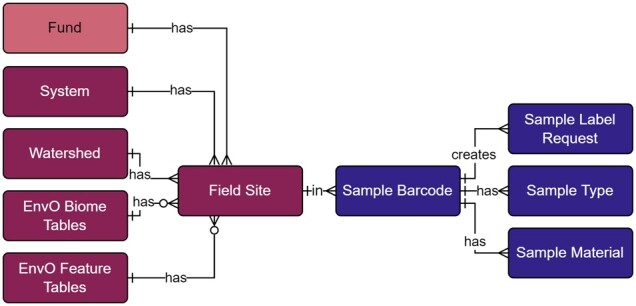
Field site and sample label modules within the metadata schema. Full schema available in Supplementary Figure S1. Sample label references the field site module through the field site table. The field site table contains all unique site IDs, where system, watershed, and EnvO tables are referenced to generate the human-readable identifier. The sample barcode table contains all unique sample labels and are generated through Celery tasks and the sample label request table. Sample type and material tables are referenced to generate the human-readable sample labels. Full descriptions of fields for the Field Site and Sample Label modules are available in Supplementary Table S2

As part of the project, we have a growing and varying set of data collection sites that are regularly resampled along with ad hoc or opportunity collection sites. Therefore, we needed the ability to tag sites with unique and human readable identifiers (Fig. 3a and b). The field site module (Fig. 4; Supplementary Table S2) represents unique field sampling locations. To ensure consistent location designators for these sites, we developed a scheme for unique site IDs. Since our deployment of the open-source database management system PostgreSQL was set up with the geospatial extension PostGIS, generating location-based aggregate summaries was significantly simplified using unique site IDs.

**Fig. 5.**
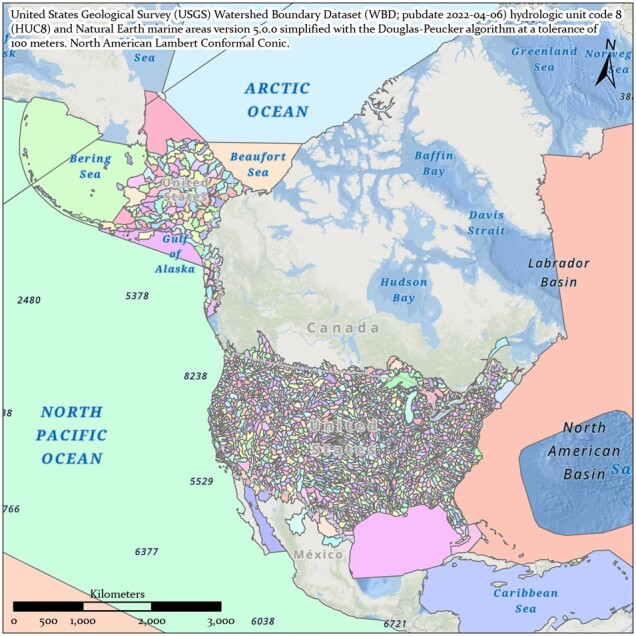
United States Geological Survey Watershed Boundary Dataset (publication date April 6, 2022) merged with Natural Earth marine areas version 5.0.0 (naturalearthdata.com) and simplified with the Douglas–Peucker algorithm at a tolerance of 100 meters in ArcGIS Pro v2.8.1

Each site ID includes a watershed region code based on the United States Geological Survey (USGS) Watershed Boundary Dataset (WBD; published April 6, 2022) hydrologic unit code 8 (HUC8) (Simley and Carswell, 2009) and the Natural Earth marine areas dataset version 5.0.0 (naturalearthdata.com) simplified with the Douglas–Peucker algorithm at a tolerance of 100 meters (Whyatt and Wade, 1988; Fig. 5).

This naming convention was adopted for consistency with the U.S. Forest Service eDNAtlas database (Young *et al.*, 2018). The system-type code in a site ID refers to what system the field site represents, for example, whether the site is within a natural or human-made impoundment (lake), a unidirectionally flowing freshwater (stream/river), a tidal transition zone between river and ocean (estuary), a fully marine site with little to no direct influence of river discharge (coast), a generally open ocean with no visible coastline (pelagic), from an aquarium (aquarium), or mock community (mock). Our legacy watershed codes for Maine are two characters in length (Fig. 3a and c), whereas the remaining >2500 watersheds and marine water bodies are three characters in length (Fig. 3b and d). The watersheds and system codes are documented respectively in the Watershed and System tables and the EnvO Biome and Feature tables were incorporated and associated with field sites to support MIxS fields (broad-scale context and local context, Yilmaz *et al.*, 2011).

These field site naming conventions were incorporated into our Sample Labels module (Fig. 4; Supplementary Table S2), where a label/barcode consists of the unique site ID, followed by the year the sample was collected, the type of material sampled (water, sediment), and a four-digit sequence (Fig. 3c and d).

#### 2.1.2 Field collection

**Fig. 6.**
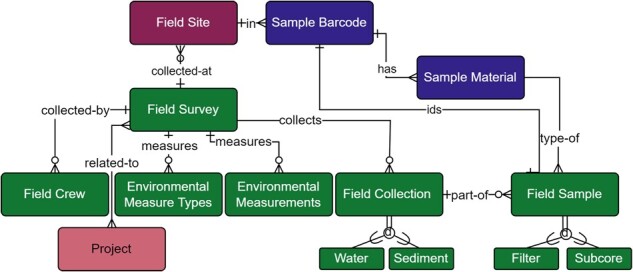
Field Survey module within the metadata schema. Full schema available in Supplementary Figure S1. The Field Survey module is related to the Field Site module through the relationship between the Field Site and Field Survey tables, where each survey is collecting materials at a Field Site. The relationship between Field Survey and Field Site is not required (open circle), however, as ‘other’ sampling sites can be used. A Field Survey may also have any number of related Field Crew, Environmental Measurements, or Field Collections (crow foot connection). The Environmental Measure Types table provides model-based choices of environmental measurements and the Environmental Measurements table stores the values of these measurements. Multiple projects can be associated with a single field survey due to the many-to-many relationship between the Field Survey and Project tables (dual crow foot connection). A Field Collection can be water or sediment, both of which may be a true collection, a mock, or a positive or negative field control, and have associated field samples that are either filters or sub-sediment/sub-core samples. Field samples are uniquely identified through their human readable sample barcode, shown by the relationship between the Field Sample and Sample Barcode table. The Field Sample table is also further described by its relationship to the Sample Material table. Full descriptions of fields for the Field Survey module are available in Supplementary Table S3

The field collection module (Fig. 6; Supplementary Table S5) incorporated wide ranging samples in both location and type. As such, the collections generated diverse data including information on environmental factors at collection sites (Environmental Measurement table), materials collected (Sample Material table), and the methods and equipment used in collection (Collection and Sample tables). General metadata included site ID, geographic location, date and time of sample collection, and field crew to provide traceability of samples and to adhere to the minimum standards of data collection across genomic databases (Field Survey and Field Crew tables; Field *et al.*, 2008; Nicholson *et al.*, 2020; Yilmaz *et al.*, 2011). Capturing environmental conditions is particularly important for downstream ecosystem and taxa-specific analyses and we met or exceeded published recommendations (Harrison *et al.*, 2019; Nicholson *et al.*, 2020). Measured environmental conditions included: Water temperature, salinity, pH, PAR1 (Photosynthetically Active Radiation Channel 1: Up looking), PAR2 (Channel 2: Down looking), turbidity, conductivity, dissolved oxygen, nitrate and nitrite, pheophytin, and chlorophyll a. Some collection locations were sampled at multiple depths in which case, each depth was associated with a corresponding set of environmental measurements and represented through the Environmental Measurement table and its relationship to the Field Survey table (Fig. 6).

Although the two primary sample types were water or sediment, our schema was developed to support the addition of types through polymorphic (or supertype/subtype hierarchy, Teorey *et al.*, 2011) relationships exemplified by the Field Collection table relationship to the types Water Collection and Sediment Collection, and the Field Sample table relationship to types Filter Sample and Subcore Sample (Fig. 6).

After collection, water was filtered using a mixed selection of filters. Filter characteristics (e.g. material type, pore size), volume filtered, length and condition of storage prior to filtering all affect the capture of DNA from environmental samples and the final yield of DNA (Kumar *et al.*, 2020). Sediment was collected via a variety of coring methods (e.g. gravity, piston, wedge) and further subdivided through a variety of methods (i.e. via scooping, use of syringe or slicing) dependent on sediment type (Pawlowski *et al.*, 2022). Collection of eDNA from sediment cores is affected by water depth, bottom substrate characteristics, equipment choice, and target organism (Pawlowski *et al.*, 2022). We interpreted field collection to include metadata up to filtration or sub-coring.

#### 2.1.3 Wet lab processing

**Fig. 7.**
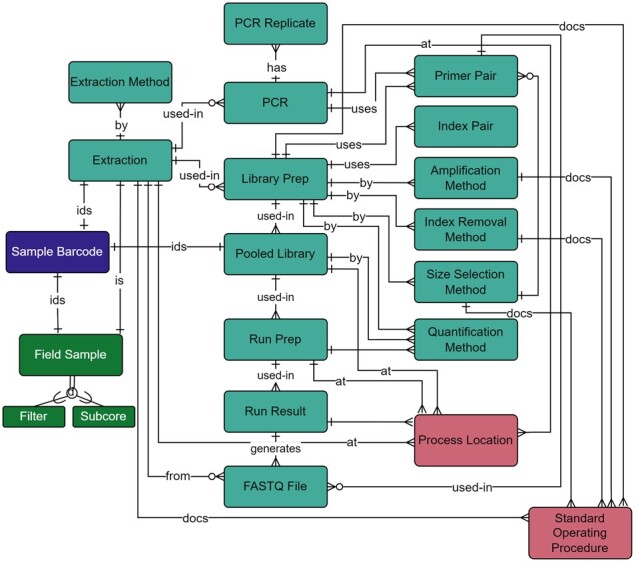
Wet Lab module within the metadata schema. Full schema available in Supplementary Figure S1. The Wet Lab module is related to the Field Survey module through the relationship between the Extraction and Field Sample tables. Extractions and pooled libraries are uniquely identified through the Sample Barcode table. qPCR and ddPCR metadata were documented in the PCR and PCR Replicate tables and through the relationship of the PCR table to the Primer Pair table. The location of wet lab preparation is documented through a table’s relationship to the Process Location Table. Associated standard operating procedures are also documented through the relationship to the Standard Operating Procedure table. All other metadata collected throughout the library preparation and sequencing steps are documented in the NGS-specific tables: Library Prep, Index Removal Method, Quantification Method, Amplification Method, Size Selection Method, and Index Pair. Library preparation primers are selected through the Primer Pair table, and proceeding NGS tables include Pooled Library, Run Prep, Run Result, and FASTQ File. Full descriptions of fields for the Wet Lab module are available in Supplementary Table S4

Field samples (filters or sub-cores) were extracted to produce testable solutions of eDNA. Within the wet lab processing module (Fig. 7; Supplementary Table S4), extraction method, final solution volume, whether DNA solution was derived from a true sample or negative control, and DNA concentration were documented, as these factors affect downstream results (Minamoto *et al.*, 2021). DNA was tested using metabarcoding (our primary next generation sequencing method), quantitative PCR (qPCR), or droplet digital PCR (ddPCR). ddPCR and qPCR use taxa-specific primers for detecting specific DNA fragments within a sample, whereas metabarcoding uses generalized primers to sequence whole groups of taxa within a sample at once.

Since extracted samples could be tested via any (or all) of these methods, our wet lab module included metadata fields for all processes (Fig. 7). Primers target a specific gene region and produce an amplicon within a known size range; these are important metadata for identifying successful amplification of a target taxonomic group (Bylemans *et al.*, 2018; Collins *et al.*, 2019). Metabarcoding includes PCR steps, library preparation, QA/QC, quantification, normalization, and pooling before sequencing can begin, all of which generate metadata that must be recorded. Of particular interest are the Primer Pair, needed for the bioinformatics pipeline, and the Index Pair. Indexes in this context are identifying tags attached to amplicons that are required to demultiplex samples after sequencing and are entered into the Index Pair table (Illumina, 2019).

To generate FASTQ files, the libraries must be sequenced (Fig. 1). The concentrations of library and standard, as well as library preparation kit details, were captured through the Run Prep and Run Results tables (Fig. 7).

#### 2.1.4 Bioinformatic analysis workflows

**Fig. 8.**
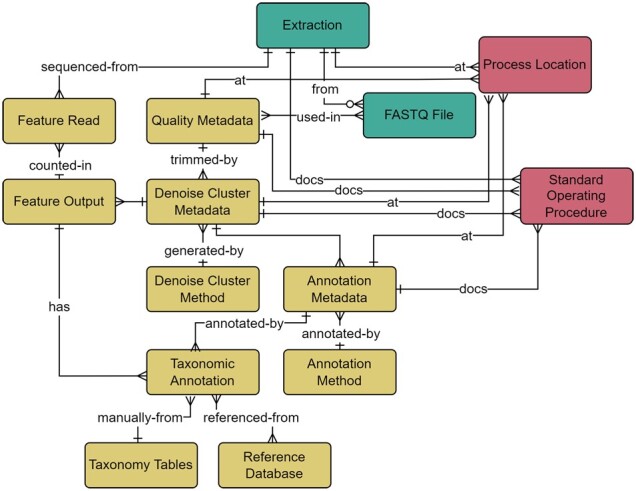
Bioinformatics module within the metadata schema. Full schema available in Supplementary Figure S1. The Bioinformatics module is related to the Wet Lab module through the relationship between the FASTQ File and Quality Metadata, where quality trimming parameters are captured and include the minimum and maximum read length, forward and reverse trim length, and sequence quality. Parameters relating to both Amplicon Sequence Variants and Operational Taxonomic Units generation methods are included within the Denoise Cluster Metadata table. Sequences generated from denoising or clustering are listed in the Feature Output table and the number of reads per sequencing run is represented in the Feature Read table. The Annotation Metadata table covers information related to the taxonomic annotation process and is related to the Taxonomic Annotation table. The Taxonomic Annotation table retains the results of any annotation method and enables the annotation of manually verified taxonomy. The Taxonomy tables (simplified here for brevity) are a set of hierarchical tables that correspond to the full taxonomic hierarchy (Domain, Kingdom, Supergroup, Phylum/Division, Class, Order, Family, Genus, and Species) and represent curated species lists that can be referenced to manually annotate verified taxonomy to a sequence through their relationship to the Taxonomic Annotation table. The location of an analysis or associated standard operating procedures are captured in relationships to the Process Location and Standard Operating Procedure tables. The Wet Lab module is further related to the Bioinformatics module through the relationship between the Feature Read and Extraction tables, where the count of reads per sample are captured. Full descriptions of fields for the Bioinformatics module are available in Supplementary Table S5

To support full transparency and traceability of analysis workflows in each sample (Wilkinson *et al.*, 2016), the bioinformatics module (Fig. 8; Supplementary Table S5) was designed to cover the full taxonomic scope, standard operating procedures (SOPs), reference database, underlying scripts, environmental files (installed software packages), and pipeline parameters. We capture fields that are standard across genomic databases (Yilmaz *et al.*, 2011), with careful consideration given to identify and include non-standard fields that could have potential impacts on annotated sequences.

As the number of bioinformatics tools and analysis methods increase, so too does the importance of capturing complete workflow steps. This is crucial, not only for comparison among samples but also to determine what parameters could impact results. The lack of consensus on best practices for processing amplicon sequence data in current literature increases the necessity of documenting biases that may arise from competing methods (O’Rourke *et al.*, 2020). Thus, the medna-metadata database sought to capture software information that could be applicable across any number of bioinformatics pipelines through a link to an environment file that records all software versions used. A standard process within a bioinformatics analysis pipeline consists of evaluating the quality of the sequences within a FASTQ file, removing primers, filtering, trimming, followed by denoising and dereplication, or clustering, before finally performing taxonomic annotations (Fig. 1).

Quality trimming can improve accuracy through assessment of read quality and removal of sequences with low-confidence base calls, or excessive ambiguous bases. Choosing strict trim values can more effectively reduce erroneous reads and artifacts, but overly conservative filtering can potentially remove too many reads, which can impose a challenge to standardizing parameter values (Piper *et al.*, 2019). Due to the subjective nature of this step, trimming parameters constitute an important meta-analysis parameter (Quality Metadata table; Piper *et al.*, 2019).

Denoising algorithms produce amplicon sequence variants (ASVs, Callahan *et al.*, 2017) through correction of single-nucleotide differences and inference of ‘actual’ sequences from sequencing noise (DADA2, Callahan *et al.*, 2016). Clustering methods, the generation of operational taxonomic units (OTUs, Blaxter *et al.*, 2005) have a similar goal of sequence reduction, but do so by using an arbitrary similarity threshold, typically 97%, whereas dereplication uses a threshold of 100%. Denoising algorithms are designed to reduce sequence artifacts from random errors in PCR amplification and sequencing using statistical models rather than arbitrary similarity thresholds to define OTUs. Methods and parameters used for deriving features (ASVs, OTUs, etc.) are captured within the Denoise Cluster Method and Metadata tables.

Additional noise can be introduced from chimeric sequences that result from incomplete primer extension during PCR. Although chimeric sequences tend to have a low number of copies, removal of these artifact sequences is important because they could be a potential source of ‘false’ sequence reads (Schnell *et al.*, 2015). The name, software version, and parameters used for chimera removal are captured within the Denoise Cluster Metadata table.

After denoising or clustering, common annotation techniques, such as Basic Local Alignment Search Tool (BLAST, Altschul *et al.*, 1990; BLAST+, Camacho *et al.*, 2009) or Multinomial Naïve Bayes (q2-feature-classifier, Bokulich *et al.*, 2018), are recorded in the Annotation Method table. Taxonomic annotation results, methods, and reference database metadata enables tracking missing or unassigned taxa within a reference database, the detection of false positives, and increases the potential to detect overlooked species (Coissac *et al.*, 2012; Schenekar *et al.*, 2020; Stoeckle *et al.*, 2020; Yilmaz *et al.*, 2011).

Collection of taxonomic names and hierarchies during taxonomic annotation is critical to understanding the biodiversity present in samples (Pappalardo *et al.*, 2021). As taxonomy and phylogeny are in perpetual flux (Beiko, 2015; Zimmermann *et al.*, 2014), collecting all taxonomic annotations from major reference databases, such as Silva (Yilmaz *et al.*, 2014), PR2 (Guillou *et al.*, 2013), and Barcode of Life Data Systems Database (http://www.barcodinglife.org, Ratnasingham and Hebert, 2007), are important for standardization across samples. Medna-metadata captures information pertaining to all Linnaean taxonomy (e.g. species, genus; Paterlini, 2007; Supplementary Table S5) and includes protist relevant groupings (i.e. supergroup and division) based on the higher-level annotations within the PR2 database (Guillou *et al.*, 2013).

#### 2.1.5 Freezer inventory tracking

**Fig. 9.**
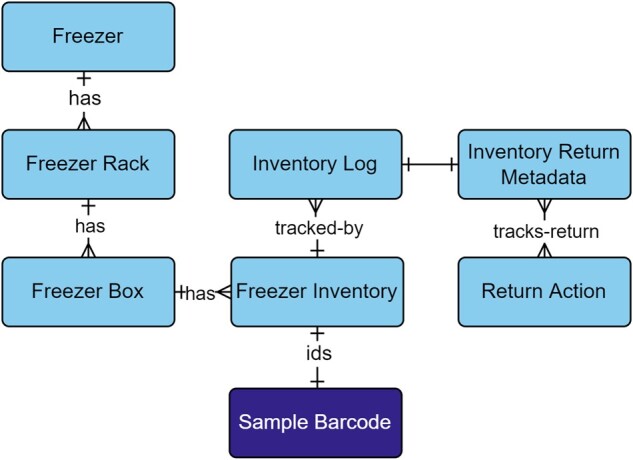
Freezer Inventory module within the metadata schema. Full schema available in Supplementary Figure S1. The Freezer Inventory module is related to samples through the Sample Barcode table. Field samples, extractions, and pooled libraries are identified by their relationship to the Sample Barcode table. Each level of the Freezer Inventory module reflects the organization of a freezer that contains racks, boxes, and sample tubes. Inventories are tracked through the Inventory Log table, which captures new additions, permanent removals, check-outs, and returns. Returned inventory logs are captured in the Inventory Return Metadata, which stores the actions that were taken on the inventory while it was checked-out and if aliquots were taken. The Return Action table provides model-based choices on actions, such as extraction, PCR, library prep, pooled library, run prep, run result, or none. Full descriptions of fields for the Freezer Inventory module are available in Supplementary Table S6

As the number of samples and storage locations increased, the ability to track the status and location of field samples, extractions, or libraries became essential to capture in the freezer inventory module (Fig. 9; Supplementary Table S6). To slow degradation of DNA in a sample, microbial community interactions, or chemical reactions, samples were stored in −80°C freezers (Kumar *et al.*, 2020). Complimentary to sample tracking, was capturing the temperature and duration of storage as they can affect results obtained from samples (Bustin *et al.*, 2009; Yilmaz *et al.*, 2011). Extractions and libraries contain solutions of DNA, small portions, or aliquots of which were periodically taken from the freezer for processing. To support on demand sample monitoring, additional attributes such as volume and level of processing were captured.

## 3 Implementation

### 3.1 Backend development

A critical consideration beyond database modeling and design was deciding on an implementation platform. Given our project goals to provide open-source and free products, we chose Django with Django’s REST Framework for our backend development. These libraries and frameworks are widely used for web application development, as evidenced by their use among other open-source projects on scientific data exchange and tracking (Anatskiy *et al.*, 2019; Androulakis *et al.*, 2011).

A major system requirement was support for real-time data ingestion from multiple sources to a central location. Django’s REST Framework enables relatively fast and secure development of an API to enable this feature. The API is fully documented using Swagger/OpenAPI 2.0 generated with the drf-yasg library. To support asynchronous and distributed task management, we incorporated Celery with the RabbitMQ message-broker.

### 3.2 API endpoints

All database tables were developed with an API endpoint. Custom API views were developed with multi-table joins for viewing related field survey information for filters (api/field_survey/survey_filters), subcores (api/field_survey/survey_subcores), environmental measurements (api/field_survey/survey_envs) and for MIxS sediment and water (api/mixs/sediment or water).

### 3.3 Frontend development

Medna-metadata’s frontend was customized from open-source frontend libraries django-material-dashboard and django-material-kit. These open-source libraries included integration with widely used frontend frameworks such as Bootstrap and Material Design. The Chart.js library was also utilized for data visualization and Leaflet for interactive web maps. Other common JavaScript libraries such as Select2 were used to create conditional and searchable dropdown menus.

### 3.4 API and frontend CRUD-permissions

Access to all database tables through the API and content in the frontend is controlled through CRUD-based permissions (create, read, update, delete), where a particular user may only perform operations based on what permissions are granted to their account. Authenticated users (or users with a login) do not automatically receive permissions and must be granted permissions by an administrator in the administrative site. Permissions can be set piecemeal, or through pre-generated permission groups available from a custom django-admin command with permissions for project admins (add, update, and view for all tables), graduate students (add, update, and view for a subset of tables), and interns (view and add for a subset of tables).

The frontend of medna-metadata has publicly (read) accessible content that includes background information on medna-metadata and Maine-eDNA, as well as project-based summaries and map views, publication lists, and a contact us page that are dynamically populated from the database. Frontend authenticated users can view standard operating procedures and API documentation. The frontend dashboard provides chart summaries, tabular views of the data, forms for adding and updating metadata for all available database tables, and data download options. Authenticated users require specific CRUD-permissions to modify content within pages on the main site or dashboard.

### 3.5 Controlled vocabulary

To enable aggregations and queries by a controlled vocabulary, we incorporated enumerated and model-based choices. Model-based choices are customizable through the administration site, whereas enumerations are hardcoded choices that are unlikely to change. Enumerations do not need to be created upon generation of the application, but model-based choices need to be populated based on a project’s needs. For example, process location may differ by project and was added as a model-based choice, whereas library layout is a controlled MIxS vocabulary that may not change often. Customization of model-based choices is restricted to administrators to limit duplication of existing choices and retain a clean and concise list.

### 3.6 MIxS MIMARKS-SURVEY

**Fig. 10.**
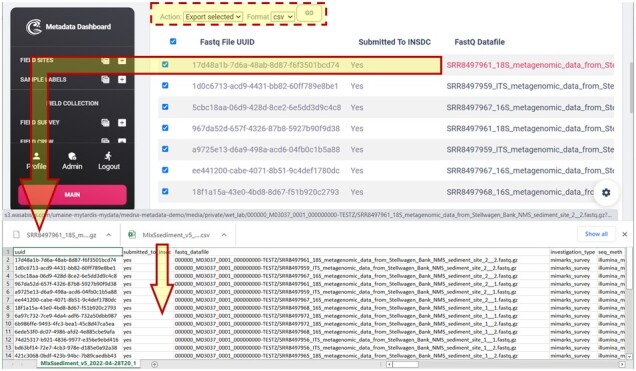
Tabular view of MIxS sediment (MIMARKS-SURVEY) mandatory (M), conditionally mandatory (C), and environmentally dependent (E) fields, and downloaded CSV and FASTQ file

One of the more challenging and important aspects of the application was providing automatically MIxS formatted outputs for mandatory (M), conditionally mandatory (C), and environmentally dependent (E) MIMARKS-SURVEY fields. Within the dashboard, medna-metadata provides tabular views of MIxS sediment and water. The tabular view also enables the user to select and export these views into a desired format, as well as download a particular FASTQ file (Fig. 10).

### 3.7 Maine-eDNA deployment

While the main branch of medna-metadata is available for projects deploying this application, use of the API with automated data ingestion from other applications will require additional customization. The following describes how we customized our medna branch for automatic data ingestion.

Because of the many sites sampled by the project, effective spatial data management was a key concern. We employed a suite of commercial GIS products from ESRI, which are widely used within academic, federal, and state sectors. For field data collection we used ESRI’s Survey123, a cross-platform data collection application with a proprietary ESRI geodatabase as the backend. Use of Survey123 eliminated a significant amount of development time that would otherwise have been required to provide a cross-platform application for field data collection.

A challenge, however, was extracting data from ESRI’s geodatabase and loading it into medna-metadata as Survey123 records were created or updated. We found Make (https://www.make.com/) to be the most user-friendly integration with Survey123, but it did come at a premium. Additional development was required within medna-metadata to transform data from Survey123 into a normalized and queryable format, since Survey123 schemas are automatically generated without normalization in mind. Our solution was to store field data in Extract, Transform, and Load (ETL) tables prior to being transformed with Django post-save signals and Celery tasks.

Another component to be cognizant of was where to store datafiles (e.g. FASTQ files, bioinformatics outputs). Within Maine-eDNA, datafiles were automatically transferred to MyTardis (Androulakis *et al.*, 2011) and the Texas Advanced Computing Center (TACC). The cloud storage service utilized for MyTardis was Wasabi S3 because there were no transfer limits, a set monthly rate, and reliable and prompt service. While MyTardis provided secure, login-based access to datafiles, there was an additional project-wide request to provide access through Google Drive. Rclone was instrumental in setting up regular synchronization between multiple virtual machines and cloud storage services. Since the datafiles were already in S3 storage via MyTardis, a combination of the django-storages library and Celery tasks were used to extract and load existing datafile locations into medna-metadata.

## 4 Discussion

### 4.1 Case study examples

As the scale of high throughput data in life sciences increases, information systems capable of managing heterogeneous data that support FAIR principles of scientific data management (Wilkinson *et al.*, 2016) are in high demand. Relational databases provide an ideal framework for representing and utilizing large-scale genomic datasets and are widely used for biological applications (Cuellar *et al.*, 2022; Di Marsico *et al.*, 2022). In large interdisciplinary projects, such as Maine-eDNA, whose goal is to collect, manipulate, store, and analyze copious amounts of information, design decisions play an important role in how the data are handled on the user side. Wang *et al.* (2019) found that while relational databases are effective at managing data, the underlying models required in-depth domain knowledge to develop robust schemas. The integration of environmental samples for the exploration of biodiversity and distribution can be achieved if metadata retrieved is formalized, curated, and standardized.

Given the scope of medna-metadata, and the amount of data captured in the underlying schema, we wanted to test the database with an initial small use-case. The case study investigated biodiversity in the New England Aquarium to evaluate eDNA detection methods for vertebrates and invertebrates (Silverbrand, 2021), through the capture of environmental measurements such as pH and salinity. Wet lab processing and sequencing parameters were documented, raw sequence reads as FASTQ files were generated, and protocol methods standard to Illumina sequencing were entered into medna-metadata. Provenance of run sequence files are important to capture because different approaches for processing raw sequences can influence diversity estimates (Reitmeier *et al.*, 2021). This study represents a fully reproducible use-case that can be efficiently retrieved and compared against other studies from environmental sampling, sequencing protocols, and analysis methods. To further demonstrate application of medna-metadata, we applied it to a published eDNA metabarcoding dataset from the Stellwagen Bank National Marine Sanctuary in the Gulf of Maine (Polinski *et al.*, 2019). These example data can be viewed from the demo deployment of medna-metadata (https://demo.metadata.maine-edna.org/main/projects/detail/15/).

Linking all underlying metadata gives access to a plethora of new information, which can be used as a driving factor to test new hypotheses, otherwise not possible if data were unlinked and unshared, and oftentimes stronger results can be obtained by using many of these data types together. The scope of the data contained within medna-metadata enables interoperability among metabarcoding studies, and because all information through the entire workflow is captured and stored, it can be used to test implications of method selection on sampling, sequencing, and analyses.

### 4.2 Expanding scope

Field sites and sample labels are dependent on the availability of watershed designators. For sites within USA, watershed designators are provided through the United States Geological Survey (USGS) Watershed Boundary Dataset (WBD, Simley and Carswell, 2009). Expanding beyond the regions covered by the WBD would be dependent on having international watershed designators, and if they did not exist, then some other source of watershed designators would need to be used.

While the initial goal of the application was to provide a project-specific resource, the application could be reimplemented at a national scale upon need. For this application to be deployed nationally, adjustments would need to be made to the field site and sample label naming conventions. Presently, the field sites and sample labels sequentially increment. For the application to be deployed as a national repository, we anticipate that there are not enough digits available for either field sites or sample labels. If there is a desire to deploy this application nationally, then replacing the sequence with a universally unique identifier (UUID) may be sufficient to support this expansion.

Although application development focused on amplicon sequencing, the metadata database schema was developed to be extendable to other cases such as shotgun sequencing. The system can still track samples that could be characterized using other sequencing methods, but the medna-metadata application would need to be modified to support sequencing workflows beyond metabarcoding.

### 4.3 Fair compatibility

Transparent and standardized data structures and analysis workflows are critical for supporting reproducibility. The ever-increasing combinations of software, software versions, and data inputs, have made reproducibility of a given field collection, wet lab procedure, or bioinformatic analysis workflow difficult when detailed information is unavailable. This is a significant concern as scientific inquiry is based on reproducibility where others can reasonably reproduce analysis results (Mesirov, 2010). Indeed, with the use of computers, such reproduction should be made easier, not more difficult.

Reproducibility is a known problem within the field, and various approaches have been suggested, many of which center around the idea of building FAIR pipelines and pipeline management systems (Barone *et al.*, 2017; Reiter *et al.*, 2021; Wratten *et al.*, 2021). To achieve FAIR standards, both data management and analysis workflow practices must be carefully documented and implemented. For example, organization of analysis through an established set of programs, which are packaged in a version-specific and platform-agnostic manner, fosters reproducibility.

Within our system, we conform to FAIR practices by providing detailed provenance through documentation. For example, for a run, we provide both the name of the pipeline used as well as the script itself, and include a list of the specific packages required. Also included is information about when, where, and by whom a particular pipeline was run. We include relevant details about the reference database used in the taxonomic assignment pipeline. In this way, we have ensured that our system is FAIR compatible, by having easily Findable pipelines, which are Accessible by the coding scripts, Interoperable in large part by the packaging of the pipelines themselves, combined by providing a detailed list of programs used, as well as largely Reusable by the strict framework in which those pipelines operate.

## Supplementary Material

btac556_supplementary_data

## Data Availability

The source code of the medna-metadata web application is hosted on GitHub (https://github.com/Maine-eDNA/medna-metadata) and licensed under the GNU General Public License v3.0. Medna-metadata is a docker-compose installable package. Documentation can be found at https://medna-metadata.readthedocs.io/en/latest/?badge=latest. The application is implemented in Python, PostgreSQL and PostGIS, RabbitMQ, and NGINX, with all major browsers supported. A demo can be found at https://demo.metadata.maine-edna.org/. The demonstration metabarcoding data were derived from a source in the public domain, available in the NCBI Sequence Read Archive (SRA) under BioProject accession number PRJNA517501 (BioSample accessions SAMN10834666–SAMN10834683). Associated sequences, count tables, and taxonomic annotations were derived from a source in the public domain, available through Zenodo at https://zenodo.org/record/6536505#.YnqPn_jMI2w. These data were originally published in Polinski *et al.*, 2019. The geospatial watershed boundary datasets were derived from a source in the public domain, available through United States Geological Survey (USGS) The National Map (publication date April 6, 2022) at https://prd-tnm.s3.amazonaws.com/index.html?prefix=StagedProducts/Hydrography/WBD/National/GDB/. The geospatial waterbody boundary datasets were derived from a source in the public domain, available through Natural Earth marine areas dataset version 5.0.0 at https://www.naturalearthdata.com/http//www.naturalearthdata.com/download/10m/physical/ne_10m_geography_marine_polys.zip.
