# Supplementary material for: medna-metadata: an open-source data management system for tracking environmental DNA samples and metadata
Source: Bioinformatics. 2022 Aug 12;38(19):4589–97. doi: 10.1093/bioinformatics/btac556 (PMC9524998; doi:10.1093/bioinformatics/btac556)
Supplement: btac556_supplementary_data [file btac556_supplementary_data.pdf]

SUPPLEMENTARY MATERIAL

medna-metadata: an open-source data management system for tracking environmental DNA samples and metadata

Kimble, M.[1,*], Allers, S.[2], Campbell, K.[1], Chen, C.[1], Jackson, L. M.[3,4], King, B. L.[2], Silverbrand, S.[5], York, G.[6] and Beard, K.[1]

[1]School of Computing and Information Science, University of Maine, Orono, ME 04469, USA, [2]Department of Molecular and Biomedical Sciences, University of Maine, Orono, ME, 04469, USA, [3]Advanced Research Computing, Security and Information Management, University of Maine, Orono, ME 04469, USA, [4]Maine EPSCoR, University of Maine, Orono, ME 04469, USA, [5]School of Marine Sciences, University of Maine, Orono, ME, 04469, USA, [6]Environmental DNA Laboratory, Coordinated Operating Research Entities, University of Maine, Orono, ME 04469, USA

*To whom correspondence should be addressed.

1 SUPPLEMENTARY FIGURES

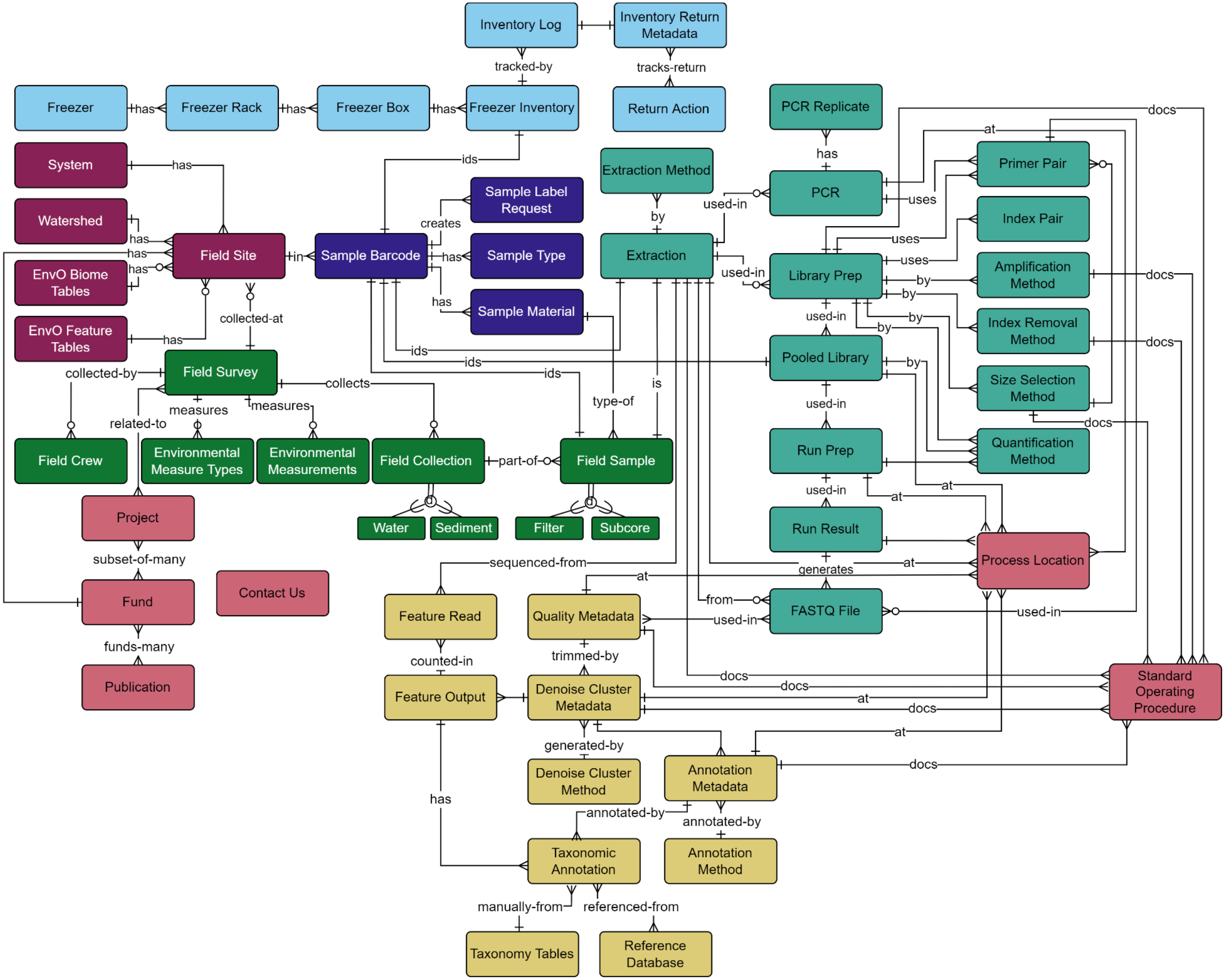

Supplementary Fig. S1: Full entity relationship diagram of relevant tables for medna-metadata.

2 SUPPLEMENTARY TABLES

Supplementary Table S1: Utility module fields: Expanded description of all fields (field, name), associated tables (table), field examples (example), related MIxS fields (MIxS), MIMARKS-SURVEY requirements (M-S), and field citations (citations) for the Utility module.

id	table	field	name	description	example	MIxS	M-S	citations
**1**	Fund	fund_code	fund code	The code for the fund.	e	project_name	M	**Field et al., 2008; Yilmaz et al., 2011**
**2**	Fund	fund_label	fund label	The label for the fund.	Maine-eDNA	project_name	M	**Field et al., 2008; Yilmaz et al., 2011**
**3**	Fund	fund_description	fund description	The description for the fund.	Maine-eDNA is a state-wide, multi-institutional initiative establishing Maine as a national leader in environmental monitoring, ecological understanding and sustainability of coastal ecosystems through research, education, and outreach.	project_name	M	**Field et al., 2008; Yilmaz et al., 2011**
**4**	Project	project_code	project code	The code for a project.	[prj_medna, prj_theme1, prj_lbb, prj_ale, prj_fisheries, prj_theme2, prj_habs, prj_spmove, prj_theme3, prj_indexsites, prj_macroint, prj_microbio, prj_commsci, ...]	project_name	M	**Field et al., 2008; Yilmaz et al., 2011**
**5**	Project	project_label	project label	The label for the project.	[Maine eDNA, Theme 1, Larval Black Box (T1), Alewife (T1), Fisheries eDNA (T1), Theme 2, Harmful algal blooms (T2), Species on the move (T2), Theme 3, Index Sites (T3), Macrosystem Integration (T3), Microbial biosensors (T3), Community Science, ...]	project_name	M	**Field et al., 2008; Yilmaz et al., 2011**
**6**	Project	project_description	project description	The description of the project; used to populate the public frontend [domain]/main/projects/.	Advance next generation eDNA-based ecological inference for cross ecosystem community structure comparison by integrating and analyzing a shared Big Data resource of eDNA data and associated spatiotemporal and environmental data amassed by all research teams.	project_name	M	**Field et al., 2008; Yilmaz et al., 2011**
**7**	Project	project_goals	project goals	The goals of the project; used to populate the public frontend [domain]/main/projects/.	Establish capacity to quantify coastal disturbances and their biogeochemical consequences (3.2.1). Advance eDNA methods for the study of estuarine microbial communities as biosensors of environmental disturbance events (3.2.2).	project_name	M	**Field et al., 2008; Yilmaz et al., 2011**
**8**	Project	fund_names	fund name	Fund associated with project. This is a foreign key to the Fund table.		project_name	M	**Field et al., 2008; Yilmaz et al., 2011**
**9**	Publication	publication_title	publication title	Title of the publication; used to populate the public frontend [domain]main/publications/.	How Does Strategic Communication Shape Transdisciplinary Collaboration? A Focus on Definitions, Audience, Expertise, and Ethical Praxis			
**10**	Publication	publication_url	publication url	The URL of the publication; used to populate the public frontend [domain]/main/publications/.	https://www.frontiersin.org/articles/10.3389/fcomm.2022.831727/full			
**11**	Publication	project_names	project names	The projects associated with the publication; This is a foreign key to the Project table. Used to populate the public frontend [domain]/main/publications/.	Team Science (T3)			
**12**	Publication	publication_authors	publication authors	The users associated with the publication; This is a foreign key to the Users table.				
**13**	Standard Operating Procedure	sop_title	SOP Title	Standard operating procedure title	Extraction protocol	sop	C, C	**Field et al., 2008**
**14**	Standard Operating Procedure	sop_url	SOP URL	Standard operating procedure URL	https://www.sop.com	sop, url	C, C	**Field et al., 2008**

id	table	field	name	description	example	MIxS	M-S	citations
**15**	Standard Operating Procedure	sop_type	SOP Type	Standard operating procedure type. This references the enumeration SopTypes.	[bioinformatics, wet_lab, field_collection, freezer_inventory]	sop	C, C	**Field et al., 2008**
**16**	Metadata Template	uuid	UUID	A universally unique identifier for each metadata template file.	f5d907a6-a814-4faa-b0da-209ba82de848			
**17**	Metadata Template	template_datafile	Template Datafile	The metadata template datafile.	WetLabDocumentation_v1.xlsx			
**18**	Metadata Template	template_type	Template Type	Template type. This references the enumeration SopTypes.	[bioinformatics, wet_lab, field_collection, freezer_inventory]	sop	C, C	**Field et al., 2008**
**19**	Metadata Template	template_version	Template Version	The version of the metadata template.				
**20**	Metadata Template	template_notes	Template Notes	Notes on the metadata template.				
**21**	Process Location	process_location_name	process location name	The name of the process location.	eDNA Laboratory (UMaine CORE)			
**22**	Process Location	affiliation	affiliation	The affiliation of the process location.	University of Maine			
**23**	Process Location	process_location_url	process location URL	The URL of the process location	https://umaine.edu/core/biotechnology/			
**24**	Process Location	phone_number	phone number	The phone number of the process location	(207) 581-2591			
**25**	Process Location	location_email_address	location email address	The general email address of the process location	um.core@maine.edu			
**26**	Process Location	point_of_contact_email_address	point of contact email address	The email address of the point of contact at the process location				
**27**	Process Location	point_of_contact_first_name	point of contact first name	The first name of the point of contact at the process location				
**28**	Process Location	point_of_contact_last_name	point of contact last name	The last name of the point of contact at the process location				
**29**	Process Location	location_notes	process location notes	Notes on the process location.				
**30**	Contact Us	full_name	full name	The name of the individual submitting to [domain]/main/contact-us/				
**31**	Contact Us	contact_email	contact email	The email of the individual submitting to [domain]/main/contact-us/				
**32**	Contact Us	contact_context	contact context	The message of the individual submitting to [domain]/main/contact-us/				
**33**	Contact Us	replied	replied	Whether the message has been replied to. This references the enumeration YesNo.	[yes, no]			
**34**	Contact Us	replied_context	replied context	The content of the response message.				
**35**	Contact Us	replied_datetime	replied datetime	The date and time the message was responded to.				

Supplementary Table S2: Field site and sample label modules fields: Expanded description of all fields (field, name), associated tables (table), field examples (example), related MIxS fields (MIxS), MIMARKS-SURVEY requirements (M-S), and field citations (citations) for the Field Site and Sample Label modules.

id	table	field	name	description	example	MIxS	M-S	citations
**1**	EnvO Biome Tables	biome_[number]_tier	biome	Biome should be treated as the descriptor of the broad ecological context of a sample (EnvO biome; http://purl.obolibrary.org/obo/ENVO_00000428)	[Area of deciduous forest, Coastal water body, Estuarine, Large lake, Large river, Large river headwater, Large river delta, Marine benthic, Marine reef, Marine salt marsh, Marine upwelling, Neritic pelagic zone, Oceanic pelagic zone, Ocean, Small lake, Small river, Temperate marginal sea, ...]	env_biome (v4), env_broad_scale (v5)	M	**Field et al., 2008; Yilmaz et al., 2011**
**2**	EnvO Biome Tables	envo_identifier	envo identifier	The identifier of the referenced EnvO feature	[ENVO:00002030]	env_feature (v4), env_local_scale (v5)	M	**Field et al., 2008; Yilmaz et al., 2011**
**3**	EnvO Biome Tables	biome_[number]_tier	Higher tier biome	If there is a higher tier biome, the related biome. This is a foreign key to a higher tier EnvO Biome table.				
**4**	EnvO Biome Tables	ontology_url	ontology url	The URL of the referenced EnvO biome	http://purl.obolibrary.org/obo/ENVO_00002030			
**5**	EnvO Feature Tables	feature_[number]_tier	feature	Compared to biome, feature is a descriptor of the more local environment a sample is collected at (EnvO geographic feature; http://purl.obolibrary.org/obo/ENVO_00000000)	[Bridge, Coastal water body, Constructed pavement, Coral reef, Estuarine tidal riverine coastal upper water column, Estuarine tidal riverine open water surface layer, Harbor, Fish hatchery, Hydrographic feature, Lake surface, Lake bed, Marine subtidal rocky reef, Nartural harbor, Ocean Time Series Station, Pier, Sea grass bed, Turbulent aquatic surface layer, ...]	env_feature (v4), env_local_scale (v5)	M	**Field et al., 2008; Yilmaz et al., 2011**
**6**	EnvO Feature Tables	envo_identifier	envo identifier	The identifier of the referenced EnvO feature	[ENVO:02000049]	env_feature (v4), env_local_scale (v5)	M	**Field et al., 2008; Yilmaz et al., 2011**

id	table	field	name	description	example	MIxS	M-S	citations
**7**	EnvO Feature Tables	feature_[number]_tier	Higher tier feature	If there is a higher tier feature, the related feature. This is a foreign key to a higher tier EnvO Feature table.				
**8**	EnvO Feature Tables	ontology_url	ontology url	The URL of the referenced EnvO feature	http://purl.obolibrary.org/obo/ENVO_02000049			
**9**	System	system_code	system code	The code of the type of system.	[L, S, E, C, P, A, M]			
**10**	System	system_label	system label	The label of the type of system.	[lake, stream/river, estuary, coast, pelagic, aquarium, mock community]			
**11**	Watershed	watershed_code	watershed code	The code used for the United States Geological Survey (USGS) Watershed Boundary Dataset (WBD) hydrologic unit code 8 (HUC8).	[AAA, AL, AR, BB, BO, BS, CO, CR, DE, EB, FI, HS, KR, LA, LK, LP, ...]	geo_loc_name	M	**Field et al., 2008; Yilmaz et al., 2011**
**12**	Watershed	watershed_label	watershed label	The label used for the United States Geological Survey (USGS) Watershed Boundary Dataset (WBD) hydrologic unit code 8 (HUC8).	[Allagash, Aroostook, Big Black River-Saint John River, Black-Ottauquechee, Becaguimec Stream-Saint John River, Contoocook, Chaudiere River, Dead, East Branch Penobscot, Fish, Headwaters Saint John River, Keswick River-Saint John River, Lower Androscoggin, Lower Kennebec, Lower Penobscot, ...]	geo_loc_name	M	**Field et al., 2008; Yilmaz et al., 2011**
**13**	Watershed	huc8	huc8	The United States Geological Survey (USGS) Watershed Boundary Dataset (WBD) hydrologic unit code 8 (HUC8).				
**14**	Watershed	states	states	The states contained within each United States Geological Survey (USGS) Watershed Boundary Dataset (WBD) hydrologic unit code 8 (HUC8).				

id	table	field	name	description	example	MIxS	M-S	citations
**15**	Watershed	lat	latitude	The centroid latitude of each United States Geological Survey (USGS) Watershed Boundary Dataset (WBD) hydrologic unit code 8 (HUC8).		lat_lon	M	**Field et al., 2008; Yilmaz et al., 2011**
**16**	Watershed	lon	longitude	The centroid longitude of each United States Geological Survey (USGS) Watershed Boundary Dataset (WBD) hydrologic unit code 8 (HUC8).		lat_lon	M	**Field et al., 2008; Yilmaz et al., 2011**
**17**	Watershed	geom	geom	The multipolygon spatial geometry field, SRID 4326	SRID=4326;MULTIPOLYGON (((-71.05364798510502 42.36019291476734, -71.04779004061702 42.361350361539, -71.04482888186529 42.36017705918428, -71.04493617022527 42.35809994318362, -71.04802607501057 42.35711685671112, -71.05233906710548 42.35713271306636, -71.05364798510502 42.36019291476734)))	lat_lon	M	**Field et al., 2008; Yilmaz et al., 2011**

id	table	field	name	description	example	MIxS	M-S	citations
**18**	Field Site	site_id	site id	A unique identifier used for when a location is resampled at some regular interval. The naming convention is pRR_SCC or pRRR_SCC, where p is the fund code, RR or RRR is the watershed code, S is the system type code, and CC is the two digit sequence. This field is generated sequentially upon creation based on fund, watershed, and system codes.	ePR_S01			
**19**	Field Site	fund	fund	A fund, or funding source associated with the site id. This is a foreign key to the Fund table.	e	project_name	M	**Field et al., 2008; Yilmaz et al., 2011**
**20**	Field Site	project	project	The project associated with the site id. This is a foreign key to the Project table.	prj_indexsites	project_name	M	**Field et al., 2008; Yilmaz et al., 2011**
**21**	Field Site	system	system	The system associated with the site id. This is a foreign key to the System table.	S			
**22**	Field Site	watershed	watershed	The watershed associated with the site id. This is a foreign key to the Watershed table.	PR	geo_loc_name	M	**Field et al., 2008; Yilmaz et al., 2011**
**23**	Field Site	general_location_name	general location name	The general location name of the site id.	Mill Brook	geo_loc_name	M	**Field et al., 2008; Yilmaz et al., 2011**
**24**	Field Site	purpose	purpose	The purpose of the site id.	index site sampling			
**25**	Field Site	envo_biome_[number]	EnvO Biome Tables	The associated EnvO biome of the site id. This represents foreign keys to each of the EnvO Biome tables.	aquatic; freshwater; freshwater river; small river	env_biome (v4), env_broad_scale (v5)	M	**Field et al., 2008; Yilmaz et al., 2011**
**26**	Field Site	envo_feature_[number]	EnvO Feature Tables	The associated EnvO feature of the site id. This represents foreign keys to each of the EnvO Feature tables.	turbulent aquatic surface layer	env_feature (v4), env_local_scale (v5)	M	**Field et al., 2008; Yilmaz et al., 2011**

id	table	field	name	description	example	MIxS	M-S	citations
**27**	Field Site	geom	geom	The point spatial geometry field, SRID 4326	SRID=4326;POINT (-68.79668000028936 44.76534999983679)	lat_lon	M	**Field et al., 2008; Yilmaz et al., 2011**
**28**	Sample Type	sample_type_code	sample type code	The code for sample types.	[fs, ex, pl]			
**29**	Sample Type	sample_type_label	sample type label	The label for sample types.	[Field Sample, Extraction, Pooled Library]			
**30**	Sample Material	sample_material_code	sample material code	The code for the type of material the sample was obtained from.	[w, s]	env_material (v4), env_medium (v5)	M	**Field et al., 2008; Yilmaz et al., 2011**
**31**	Sample Material	sample_material_label	sample material label	The label for the type of material the sample was obtained from.	[water, sediment]	env_material (v4), env_medium (v5)	M	**Field et al., 2008; Yilmaz et al., 2011**
**32**	Sample Label Request	site_id	site id	The site id associated with the sample label request. This is a foreign key to the Field Site table.	ePR_S01			
**33**	Sample Label Request	sample_material	sample material	The sample material associated with the sample label request. This is a foreign key to the Sample Material table.	w	env_material (v4), env_medium (v5)	M	**Field et al., 2008; Yilmaz et al., 2011**
**34**	Sample Label Request	sample_type	sample type	The sample type associated with the sample label request. This is a foreign key to the Sample Type table.	fs			
**35**	Sample Label Request	sample_year	sample year	The sample year associated with the sample label request.	2021			
**36**	Sample Label Request	purpose	purpose	The purpose of the sample label request.	index sites sampling			
**37**	Sample Label Request	req_sample_label_num	requested sample number	The total requested number of sample labels.	60			
**38**	Sample Barcode	sample_label_request	sample label request	The sample label request associated with the sample barcode.				

id	table	field	name	description	example	MIxS	M-S	citations
**39**	Sample Barcode	sample_barcode_id	sample barcode id	The sample barcode. The naming convention is pRR_SCC_YYm_CCCC where p is the fund or project code, RR is the region code, S is the system type code, CC is the two digit sequence, YY is the two digit collection year, m is the sample material code, and CCCC is the four digit sequence.	ePR_S01_21w_0001			
**40**	Sample Barcode	in_freezer	in freezer	Boolean indication of whether the sample is in a freezer. This references the enumeration YesNo.	[yes, no]			
**41**	Sample Barcode	site_id	site id	The site id associated with the sample barcode.	ePR_S01			
**42**	Sample Barcode	sample_material	sample material	The material associated with the sample barcode. This is a foreign key to the Sample Material table.	w	env_material (v4), env_medium (v5)		**Field et al., 2008; Yilmaz et al., 2011**
**43**	Sample Barcode	sample_type	sample type	The sample type associated with the sample barcode. This is a foreign key to the Sample Type table.	fs			
**44**	Sample Barcode	sample_year	sample year	The sample year associated with the sample barcode.	2021			
**45**	Sample Barcode	purpose	purpose	The purpose of the sample barcode.	index sites sampling			

Supplementary Table S3: Field survey module fields: Expanded description of all fields (field, name), associated tables (table), field examples (example), related MIxS fields (MIxS), MIMARKS-SURVEY requirements (M-S), and field citations (citations) for the Field Survey module.

id	table	field	name	description	example	MIxS	M-S	citations
**1**	Field Survey	survey_global_id	survey global id	ESRI file geodatabase global identifier for field survey record.				**Nicholson et al., 2019**
**2**	Field Survey	username	username	Username record was submitted under. This is a foreign key to the Users table.				**Nicholson et al., 2019**
**3**	Field Survey	survey_datetime	survey datetime	Survey date and time.				**Nicholson et al., 2019**
**4**	Field Survey	project_ids	project ids	Each affiliated project. This is a foreign key to the Project table.	[Maine eDNA, Theme 1, Larval Black Box (T1), Alewife (T1), Fisheries eDNA (T1), Theme 2, Harmful algal blooms (T2), Species on the move (T2), Theme 3, Index Sites (T3), Macrosystem Integration (T3), Microbial biosensors (T3), Community Science]	project_name	M	**Field et al., 2008; Yilmaz et al., 2011; Nicholson et al., 2019**
**5**	Field Survey	supervisor	supervisor	Designated supervisor of user submitting the record. This is a foreign key to the Users table.				**Nicholson et al., 2019**
**6**	Field Survey	recorder_fname	recorder first name	Survey recorder first name.				**Nicholson et al., 2019**
**7**	Field Survey	recorder_lname	recorder last name	Survey recorder last name.				**Nicholson et al., 2019**
**8**	Field Survey	site_id	site id	Unique survey site identifier. This is a foreign key to the Field Site table.	[eLP_E01, eLP_E02, eLP_E04, eLP_L02, eLP_S01, eMC_C01, eMC_C03, eMC_C04, eMC_C05, eMC_C06, eMC_C07, ...]			**Minamoto et al., 2021**
**9**	Field Survey	site_id_other	other site id	Other site identifiers. I.e., from another project.				

id	table	field	name	description	example	MIxS	M-S	citations
**10**	Field Survey	site_name	site name	General descriptor for the survey site.	[Hampden Marina Penobscot River, Whole Oceans site Penobscot River, Bucksport Marina, Chemo Pond, Blackman Stream, Hurricane Island, Winter Harbor Marina, Port Clyde Marina, Bucks Harbor, Downeast Institute (DEI), Andrew's Island Penobscot Bay, ...]	geo_loc_name	M	**Field et al., 2008; Yilmaz et al., 2011; Minamoto et al., 2021; Nicholson et al., 2019**
**11**	Field Survey	env_obs_turbidit y	observed turbidity	General observation of water turbidity. This references the enumeration TurbidTypes.	[none, low, medium, high]	turbidity	X	**Yilmaz et al., 2011; Barnes et al., 2014; Harrison et al., 2019; Nicholson et al., 2019**
**12**	Field Survey	env_obs_precip	observed precipitation	General observation of precipitation. This references the enumeration PrecipTypes.	[none, drizzle, light_rain, mod_rain, heavy_rain, hail, sleet, light_snow, mod_snow, heavy_snow]	atmospheric_data	X	**Yilmaz et al., 2011; Harrison et al., 2019; Nicholson et al., 2019**
**13**	Field Survey	env_obs_wind_s peed	observed wind speed	General observation of wind speed. This references the enumeration WindSpeeds.	[none, light_wind, mod_wind, strong_wind]	atmospheric_data	X	**Yilmaz et al., 2011; Harrison et al., 2019; Nicholson et al., 2019**
**14**	Field Survey	env_obs_cloud_ cover	observed cloud cover	General observation of cloud cover. This references the enumeration CloudCovers.	[none, partly_cloudy, full_cloudy]	atmospheric_data	X	**Yilmaz et al., 2011; Barnes et al., 2014; Harrison et al., 2019; Nicholson et al., 2019**

id	table	field	name	description	example	MIxS	M-S	citations
**15**	Field Survey	env_biome	EnvO biome	The major environmental system the sample or specimen came from. The systems identified should have a coarse spatial grain, to provide the general environmental context of where the sampling was done (e.g. were you in the desert or a rainforest?). We recommend using subclasses of ENVO's biome class: http://purl.obolibrary.org/obo/ENVO_00000428. Format (one term): termLabel [termID], Format (multiple terms): termLabel [termID]|termLabel [termID]|termLabel [termID] (GSC Minimum Information about any Sequence; MIxS v5).	[Area of deciduous forest, Coastal water body, Estuarine, Large lake, Large river, Large river headwater, Large river delta, Marine benthic, Marine reef, Marine salt marsh, Marine upwelling, Neritic pelagic zone, Oceanic pelagic zone, Ocean, Small lake, Small river, Temperate marginal sea, ...]	env_biome (v4), env_broad_scale (v5)	M	**Field et al., 2008; Yilmaz et al., 2011; Harrison et al., 2019; Nicholson et al., 2019**
**16**	Field Survey	env_feature	EnvO feature	The entity or entities which are in the sample or specimen's local vicinity and which you believe have significant causal influences on the sample or specimen. Please use terms that are present in ENVO and which are of smaller spatial grain than your entry for env_broad_scale. Format (one term): termLabel [termID]; Format (multiple terms): termLabel [termID]|termLabel [termID]|termLabel [termID] (GSC Minimum Information about any Sequence; MIxS v5).	[Bridge, Coastal water body, Constructed pavement, Coral reef, Estuarine tidal riverine coastal upper water column, Estuarine tidal riverine open water surface layer, Harbor, Fish hatchery, Hydrographic feature, Lake surface, Lake bed, Marine subtidal rocky reef, Nartural harbor, Ocean Time Series Station, Pier, Sea grass bed, Turbulent aquatic surface layer, ...]	env_feature (v4), env_local_scale (v5)	M	**Field et al., 2008; Yilmaz et al., 2011; Harrison et al., 2019; Nicholson et al., 2019**

id	table	field	name	description	example	MIxS	M-S	citations
**17**	Field Survey	env_material	EnvO material	The environmental material or materials (pipe separated) immediately surrounding the sample or specimen prior to sampling (GSC Minimum Information about any Sequence; MIxS v5). This references the enumeration EnvoMaterials.	[Soil, Water, Air]	env_material (v4), env_medium (v5)	M	**Field et al., 2008; Yilmaz et al., 2011; Harrison et al., 2019; Nicholson et al., 2019**
**18**	Field Survey	env_notes	environmental notes	Survey notes.				
**19**	Field Survey	env_measure_mode	measure mode	Mode through which sample was collected. This references the enumeration MeasureModes.	[On foot, Boat]			**Harrison et al., 2019; Nicholson et al., 2019**
**20**	Field Survey	env_boat_type	boat type	If measure mode was by boat, type of boat used to collect sample.	[Motorboat, Kayak, Canoe]			**Harrison et al., 2019; Nicholson et al., 2019**
**21**	Field Survey	env_bottom_depth	bottom depth	Water column, or absolute bottom depth at collection location in meters (m).		tot_depth_water_col	X	**Field et al., 2008; Yilmaz et al., 2011; Harrison et al., 2019; Nicholson et al., 2019**
**22**	Field Survey	measurements_taken	measurements taken	Booleain indication of whether environmental measurements were taken. This references the enumeration YesNo.	[yes, no]			
**23**	Field Survey	survey_complete	survey complete	Boolean indication of whether survey is complete. This references the enumeration YesNo.	[yes, no]			**Harrison et al., 2019; Nicholson et al., 2019**
**24**	Field Survey	qa_editor	QA editor	If the survey is complete, the username that performed the quality assurance check. This is a foreign key to the Users table.				**Harrison et al., 2019; Nicholson et al., 2019**
**25**	Field Survey	qa_datetime	QA datetime	If the survey is complete, the date and time of the quality assurance check.				**Harrison et al., 2019; Nicholson et al., 2019**

id	table	field	name	description	example	MIxS	M-S	citations
**26**	Field Survey	qa_initial	QA initial	If the survey is complete, the initials of the person that performed the quality assurance check.				**Harrison et al., 2019; Nicholson et al., 2019**
**27**	Field Survey	gps_alt	GPS captured altitude	The altitude captured by the device submitting the survey record in meters (m).		alt_elev, alt, elev	E	**Field et al., 2008; Yilmaz et al., 2011; Harrison et al., 2019; Nicholson et al., 2019**
**28**	Field Survey	gps_horacc	GPS captured horizontal accuracy	The horizontal accuracy captured by the device submitting the survey record in meters (m).				**Harrison et al., 2019; Nicholson et al., 2019**
**29**	Field Survey	gps_vertacc	GPS captured vertical accuracy	The vertical accuracy captured by the device submitting the survey record in meters (m).				**Harrison et al., 2019; Nicholson et al., 2019**
**30**	Field Survey	geom	geom	The point spatial geometry field, SRID 4326.	SRID=4326;POINT (-68.79668000028936 44.76534999983679)			**Harrison et al., 2019; Nicholson et al., 2019**
**31**	Field Crew	crew_global_id	crew global id	ESRI file geodatabase global identifier for field crew record.				**Nicholson et al., 2019**
**32**	Field Crew	crew_fname	crew first name	Field crew first name.				**Nicholson et al., 2019**
**33**	Field Crew	crew_lname	crew last name	field crew last name.				**Nicholson et al., 2019**
**34**	Field Crew	survey_global_id	survey global id	ESRI file geodatabase global identifier for related field survey record. This is a foreign key to the Field Survey table.				**Nicholson et al., 2019**
**35**	Env Measure Type	env_measure_type_code	env measurement type code	The code of the environmental measurement.	[env_flow, env_water_temp, env_salinity, env_ph, env_par1, env_par2, env_turbidity, env_conductivity, env_do, env_pheophytin, env_chla, env_no3no2, env_no2, env_nh4, env_phosphate, env_substrate, env_labdatetime, env_dnotes, ...]			

id	table	field	name	description	example	MIxS	M-S	citations
**36**	Env Measure Type	env_measure_type_label	env measurement type label	The label of the environmental measurement.	[flow, water temperature, salinity, pH, PAR1, PAR2, turbidity, conductivity, dissolved oxygen, ...]			
**37**	Environmental Measurement	env_global_id	environmental global id	ESRI file geodatabase global identifier for environmental measurement record.				**Harrison et al., 2019**
**38**	Environmental Measurement	env_measure_datetime	measurement datetime	The date and time environmental conditions were measured.				**Harrison et al., 2019**
**39**	Environmental Measurement	env_measure_depth	measurement depth	Depth environmental conditions were measured at in meters (m).				**Harrison et al., 2019**
**40**	Environmental Measurement	env_instrument	instruments used	Instruments used to measure environmental conditions. This references the enumeration EnvInstruments.	[CTD, YSI, Secchi Disk, Niskin]			**Harrison et al., 2019**
**41**	Environmental Measurement	env_ctd_filename	CTD file name	If a CTD was used to measure environmental conditions, the file name the CTD measurements were saved under.				**Harrison et al., 2019**
**42**	Environmental Measurement	env_ctd_notes	CTD notes	If a CTD was used, any notes on the CTD.				
**43**	Environmental Measurement	env_ysi_filename	YSI file name	If a YSI was used to measure environmental conditions, the file name the YSI measurements were saved under.				**Harrison et al., 2019**
**44**	Environmental Measurement	env_ysi_model	YSI model	The YSI model used to measure environmental conditions. This references the enumeration YsiModels.	[EXO2, EXO HANDHELD, ProDSS]			**Harrison et al., 2019**
**45**	Environmental Measurement	env_ysi_sn	YSI serial number	The serial number of the YSI used to measure environmental conditions.				**Harrison et al., 2019**
**46**	Environmental Measurement	env_ysi_notes	YSI notes	If a YSI was used, any notes on the YSI.				

id	table	field	name	description	example	MIxS	M-S	citations
**47**	Environmental Measurement	env_secchi_depth	secchi depth	The last depth the secchi depth could be observed at in meters (m).		turbidity	X	**Field et al., 2008; Yilmaz et al., 2011; Barnes et al., 2014; Harrison et al., 2019**
**48**	Environmental Measurement	env_secchi_notes	secchi notes	If a secchi disk was used, any notes on the secchi depth.				
**49**	Environmental Measurement	env_niskin_number	niskin number	If environmental conditions were measured from samples taken from a Niskin, the associated niskin number.				**Harrison et al., 2019**
**50**	Environmental Measurement	env_niskin_notes	niskin notes	If a niskin was used, any notes on the niskin.				
**51**	Environmental Measurement	env_inst_other	other instruments	Other instruments used to collect environmental measurements.				**Harrison et al., 2019**
**52**	Environmental Measurement	env_measurement	environmental measurements	List of measured environmental conditions. This is a foreign key to the Env Measure Type table.	[Flow, Water Temp, Salinity, pH, PAR1, PAR2, Turbidity, Cond, DO, Pheo, Chl-a, NO3NO2, NO2, NH4, PO4, Substrate]			**Harrison et al., 2019**
**53**	Environmental Measurement	env_flow_rate	flow rate	Water current, or flow rate measured in meters per second (m/s).		water_current	X	**Yilmaz et al., 2011; Harrison et al., 2019**
**54**	Environmental Measurement	env_water_temp	water temperature	Water temperature measured in degrees Celsius (°C).		temp	X	**Yilmaz et al., 2011; Barnes et al., 2014; Barnes and Turner 2016; Strickler et al., 2015; Harrison et al., 2019**
**55**	Environmental Measurement	env_salinity	salinity	Salinity measured in practical salinity units (PSU).		salinity	X	**Yilmaz et al., 2011; Barnes et al., 2014; Harrison et al., 2019**
**56**	Environmental Measurement	env_ph_scale	pH scale	Measured pH scale.		ph	X	**Yilmaz et al., 2011; Barnes et al., 2014; Harrison et al., 2019**
**57**	Environmental Measurement	env_par1	PAR1	Photosynthetically Active Radiation (Channel 1: Up looking) measured in micromoles per second per square meter (µmoles/sec/m²).				**Barnes et al., 2014; Harrison et al., 2019**

id	table	field	name	description	example	MIxS	M-S	citations
**58**	Environmental Measurement	env_par2	PAR2	Photosynthetically Active Radiation (Channel 2: Down looking) measured in micromoles per second per square meter (µmoles/sec/m²).		down_par	X	**Yilmaz et al., 2011; Barnes et al., 2014; Harrison et al., 2019**
**59**	Environmental Measurement	env_turbidity	turbidity	Water turbidity measured in Formazin Nephelometric Unit (FNU).		turbidity	X	**Yilmaz et al., 2011; Barnes et al., 2014; Harrison et al., 2019**
**60**	Environmental Measurement	env_conductivity	conductivity	Conductivity measured in microsiemens per centimeter (µS/cm).		conduc	X	**Yilmaz et al., 2011; Barnes et al., 2014; Harrison et al., 2019**
**61**	Environmental Measurement	env_do	dissolved oxygen	Dissolved oxygen measured in milligram per liter (mg/L).		diss_oxygen	X	**Yilmaz et al., 2011; Barnes et al., 2014; Harrison et al., 2019**
**62**	Environmental Measurement	env_pheophytin	pheophytin	Pheophytin measured in micrograms per liter (µg/L).				**Harrison et al., 2019**
**63**	Environmental Measurement	env_chla	chlorophyll a	Chlorophyll-a measured in micrograms per liter (µg/L).		chlorophyll	X	**Yilmaz et al., 2011; Harrison et al., 2019**
**64**	Environmental Measurement	env_no3no2	nitrate and nitrite	Nitrate and nitrite measured in µM.		nitro	X	**Yilmaz et al., 2011; Harrison et al., 2019**
**65**	Environmental Measurement	env_no2	nitrite	Nitrite measured in µM.		nitrite	X	**Yilmaz et al., 2011; Harrison et al., 2019**
**66**	Environmental Measurement	env_nh4	ammonium	Ammonium measured in µM.		ammonium	X	**Yilmaz et al., 2011; Harrison et al., 2019**
**67**	Environmental Measurement	env_phosphate	phosphate	Phosphate measured in µM.		phosphate	X	**Yilmaz et al., 2011; Harrison et al., 2019**
**68**	Environmental Measurement	env_substrate	bottom substrate	Observed bottom substrate. This references the enumeration BottomSubstrates.	[Pebble, Cobble, Boulder, Silt, Clay, Organic]			**Barnes et al., 2014; Harrison et al., 2019**
**69**	Environmental Measurement	env_lab_datetime	lab datetime	If environmental conditions require lab processing to obtain measurements, the date and time of processing.				**Harrison et al., 2019**
**70**	Environmental Measurement	env_measure_notes	measurement notes	Environmental measurement notes.				

id	table	field	name	description	example	MIxS	M-S	citations
71	Environmental Measurement	survey_global_id	survey global id	ESRI file geodatabase global identifier for related field survey record. This is a foreign key to the Field Survey table.				**Harrison et al., 2019**
72	Field Collection	collection_global_id	collection global id	ESRI file geodatabase global identifier for field collection record.				**Minamoto et al., 2021**
73	Field Collection	collection_type	collection type	The type of sample material being collected. This references the enumeration CollectionTypes.	[Water, Sediment]			**Minamoto et al., 2021**
74	Field Collection	survey_global_id	survey global id	ESRI file geodatabase global identifier for related field survey record. This is a foreign key to the Field Survey table.				**Minamoto et al., 2021**
75	Water Collection	field_collection	field collection	The associated field collection. This is a foreign key to the Field Collection table.				**Minamoto et al., 2021; Rees et al., 2014**
76	Water Collection	water_control	water control	Boolean indication of whether the collection is a control. This references the enumeration YesNo.	[yes, no]			**Bustin et al., 2009; Minamoto et al., 2021; Rees et al., 2014**
77	Water Collection	water_control_type	water control type	If the collection is a control, the type of control. If the extraction is a control, the type of control. A field control is deionized (DI) water exposed to air in the field. A lab control is a blank filter that was placed on the filter apparatus with fresh DI water (not the same DI water as the field control). An extraction control is a dry, unused filter that is put into a tube and extracted. A No Template Control is DNA free water used in PCR. A Mock Community is a positive control comprised of pre-selected specimens. An Environmental Standard is a positive control that is derived from a single water collection taken at a distinct location and time. This references the enumeration ControlTypes.	[field, lab, extraction, no_template_control, mock_community, env_standard, ...]			**Minamoto et al., 2021; Rees et al., 2014**
78	Water Collection	water_vessel_label	water vessel label	The label written on the collection vessel.				**Minamoto et al., 2021; Rees et al., 2014**

id	table	field	name	description	example	MIxS	M-S	citations
**79**	Water Collection	water_collect_datetime	water collection datetime	The date and time of the collection.		collection_date	M	**Yilmaz et al., 2011; Yamanaka et al., 2016; Minamoto et al., 2021; Rees et al., 2014**
**80**	Water Collection	water_collect_depth	water collection depth	The vertical distance below surface that the water was collected at, measured in meters (m).		depth	E	**Yilmaz et al., 2011; Minamoto et al., 2021; Rees et al., 2014**
**81**	Water Collection	water_collect_mode	collection mode	The mode through which water was collected. This references the enumeration WaterCollectionModes.	[hand, niskin_handtoss, niskin_array]	samp_collect_device	C	**Yilmaz et al., 2011; Minamoto et al., 2021; Rees et al., 2014**
**82**	Water Collection	water_niskin_number	niskin number	If water was collected via a niskin, the niskin the water was collected from.				
**83**	Water Collection	water_niskin_vol	niskin sample volume	If water was collected via a niskin, the volume in milliliters (mL).				
**84**	Water Collection	water_vessel_vol	water vessel volume	The volume of the final water collection vessel in milliliters (mL).		samp_size	C	**Bustin et al., 2009; Yilmaz et al., 2011; Minamoto et al., 2021**
**85**	Water Collection	water_vessel_material	water vessel material	The material of the final water collection vessel.	plastic	samp_collect_device	C	**Yilmaz et al., 2011; Minamoto et al., 2021**
**86**	Water Collection	water_vessel_color	water vessel color	The color of the final water collection vessel.	amber	samp_collect_device	C	**Yilmaz et al., 2011; Minamoto et al., 2021**
**87**	Water Collection	water_collect_notes	water collection notes	Notes on the water collection.				
**88**	Water Collection	was_filtered	filtered	Boolean indication of whether the water collection was filtered. This references the enumeration YesNo.		samp_mat_process	C	**Yilmaz et al., 2011; Minamoto et al., 2021**
**89**	Sediment Collection	field_collection	field collection	The associated field collection. This is a foreign key to the Field Collection table.				**Pawlowski et al., 2022**

id	table	field	name	description	example	MIxS	M-S	citations
**90**	Sediment Collection	core_control	core control	Boolean indication of whether the collection is a control. This references the enumeration YesNo.	[yes, no]			**Bustin et al., 2009; Pawlowski et al., 2022**
**91**	Sediment Collection	core_label	core label	The label written on the collection vessel.				**Pawlowski et al., 2022**
**92**	Sediment Collection	core_datetime_st art	core datetime start	The start date and time of the collection.		collection_date	M	**Yilmaz et al., 2011; Pawlowski et al., 2022**
**93**	Sediment Collection	core_datetime_e nd	core datetime end	The end date and time of the collection.		collection_date	M	**Yilmaz et al., 2011; Pawlowski et al., 2022**
**94**	Sediment Collection	core_method	corer method	Method through which sediment core was collected. This references the enumeration SedimentMethods.	[gravity, piston, wedge, van veen grab, other]	samp_collect_device	C	**Yilmaz et al., 2011; Pawlowski et al., 2022**
**95**	Sediment Collection	core_method_ot her	other corer method	If method is other, the other method through which sediment core was collected.				
**96**	Sediment Collection	core_collect_dep th	core collection depth	The vertical distance below local surface, where depth is measured from the sediment or soil surface and in meters (m).		depth	E	**Yilmaz et al., 2011; Pawlowski et al., 2022**
**97**	Sediment Collection	core_length	core length (cm)	Length of collected sediment core in centimeters (cm).		samp_size	C	**Bustin et al., 2009; Yilmaz et al., 2011; Pawlowski et al., 2022**
**98**	Sediment Collection	core_diameter	core diameter (cm)	Diameter of collected sediment core in centimeters (cm).		samp_size	C	**Bustin et al., 2009; Yilmaz et al., 2011; Pawlowski et al., 2022**
**99**	Sediment Collection	core_notes	core notes	Core collection notes.				
**100**	Sediment Collection	subcores_taken	subcored	Boolean indication of whether the sediment collection was subcored. This references the enumeration YesNo.	[yes, no]	samp_mat_process	C	**Yilmaz et al., 2011; Pawlowski et al., 2022**
**101**	Field Sample	sample_global_i d	sample global id	ESRI file geodatabase global identifier for field sample record.				

id	table	field	name	description	example	MIxS	M-S	citations
**102**	Field Sample	field_sample_barcode	field sample barcode	Barcode to uniquely identify each field sample. This is a foreign key to the Sample Barcode table.				
**103**	Field Sample	sample_material	sample material	The type of material the sample was obtained from. This is a foreign key to the Sample Material table.	[water, sediment]			
**104**	Field Sample	is_extracted	extracted	Boolean indication of whether the field sample was extracted. This references the enumeration YesNo.	[yes, no]	samp_mat_process	C	**Yilmaz et al., 2011**
**105**	Field Sample	collection_global_id	collection global id	ESRI file geodatabase global identifier for related field collection record. This is a foreign key to the Field Collection table.				
**106**	Filter Sample	field_sample	field sample	The associated field sample. This is a foreign key to the Field Sample table.				
**107**	Filter Sample	filter_location	filter location	Location of filtration. This references the enumeration FilterLocations.	[in_field, in_lab]			
**108**	Filter Sample	is_prefilter	prefilter	Boolean indication of whether the water collection was prefiltered with a coarse nitex prefilter prior to filtration with a finer filter, such as supor or glass fiber filter. This references the enumeration YesNo.	[yes, no]	samp_mat_process	C	**Yilmaz et al., 2011**
**109**	Filter Sample	filter_fname	filterer first name	Filterer first name.				
**110**	Filter Sample	filter_lname	filterer last name	Filterer last name.				
**111**	Filter Sample	filter_sample_label	filter sample label	The label written on the filter sample.				
**112**	Filter Sample	filter_datetime	filter datetime	Date and time of water collection filtration.		collection_date	M	Yilmaz et al., 2011; Yamanaka et al., 2016

id	table	field	name	description	example	MIxS	M-S	citations
**113**	Filter Sample	filter_protocol	filter protocol	A literature reference, electronic resource or a standard operating procedure (SOP) that describes the field sample collection method. This is a foreign key to the Standard Operating Procedures table.				
**114**	Filter Sample	filter_method	filter method	The method used to filter a water collection. This references the enumeration FilterMethods.	[vacuum, gravity, peristaltic, other]	samp_mat_process	C	**Yilmaz et al., 2011; Rees et al., 2014**
**115**	Filter Sample	filter_method_other	other filter method	If method is other, the other method through which water was filtered.				
**116**	Filter Sample	filter_vol	filter volume	The total volume of water filtered in milliliters (mL).		samp_vol_we_dna_ext	X	**Bustin et al., 2009; Yilmaz et al., 2011; Rees et al., 2014**
**117**	Filter Sample	filter_type	filter type	The type of filter used to filter a water collection. This references the enumeration FilterTypes.	[Nitex, Glass Fiber Filter (GF/F), Supor, Cellulose Nitrate (CN), Other]	samp_mat_process	C	**Yilmaz et al., 2011; Lacoursière-Roussel et al., 2016**
**118**	Filter Sample	filter_type_other	other filter type	If type is other, the other type used to filter a water collection.		samp_mat_process	C	**Yilmaz et al., 2011; Lacoursière-Roussel et al., 2016**
**119**	Filter Sample	filter_pore	filter pore	The pore size of the filter in microns (µm). Typically, Nitex are 80 µm, Glass Fiber Filter (GF/F) are 0.7 µm, Supor are 0.2 µm, and Cellulose Nitrate (CN) are 0.7 µm.	[0.2, 0.7, 80]	samp_mat_process	C	**Yilmaz et al., 2011; Lacoursière-Roussel et al., 2016**
**120**	Filter Sample	filter_size	filter size	The diameter of the filter in milimeters (mm). Typically, Nitex, Glass Fiber Filter (GF/F), Supor, and Cellulose Nitrate (CN) are 47 mm.	47	samp_mat_process	C	**Yilmaz et al., 2011; Lacoursière-Roussel et al., 2016**
**121**	Filter Sample	filter_notes	filter notes	Filter notes.				
**122**	Subcore Sample	field_sample	field sample	The associated field sample. This is a foreign key to the Field Sample table.				**Pawlowski et al., 2022**
**123**	Subcore Sample	subcore_fname	subcorer first name	Subcorer first name.				**Pawlowski et al., 2022**
**124**	Subcore Sample	subcore_lname	subcorer last name	Subcorer last name.				**Pawlowski et al., 2022**
**125**	Subcore Sample	subcore_sample_label	subcore sample label	The subcore label.				

id	table	field	name	description	example	MIxS	M-S	citations
**126**	Subcore Sample	subcore_protocol	subcore protocol	A literature reference, electronic resource or a standard operating procedure (SOP) that describes the field sample collection method. This is a foreign key to the Standard Operating Procedures table.				
**127**	Subcore Sample	subcore_method	subcore method	The method used to subset a sediment. This references the enumeration SubSedimentMethods.	[slices, syringe, other]	samp_mat_process	C	**Yilmaz et al., 2011; Pawlowski et al., 2022**
**128**	Subcore Sample	subcore_method_other	other subcore method	If method is other, the other method used to subset sediment.				**Pawlowski et al., 2022**
**129**	Subcore Sample	subcore_datetime_start	subcore datetime start	The start date and time the sediment core was subcored.		collection_date	M	**Yilmaz et al., 2011; Pawlowski et al., 2022**
**130**	Subcore Sample	subcore_datetime_end	subcore datetime end	The end date and time the sediment core was subcored.		collection_date	M	**Yilmaz et al., 2011; Pawlowski et al., 2022**
**131**	Subcore Sample	subcore_number	subcore number	The total number of subsets obtained from sediment.	60			**Pawlowski et al., 2022**
**132**	Subcore Sample	subcore_length	subcore length	The length (or thickness) of each subcore in centimeters (cm).		samp_vol_we_dna_ext	X	**Bustin et al., 2009; Yilmaz et al., 2011; Pawlowski et al., 2022**
**133**	Subcore Sample	subcore_diameter	subcore diameter	The diameter of each subcore in centimeters (cm).		samp_vol_we_dna_ext	X	**Bustin et al., 2009; Yilmaz et al., 2011; Pawlowski et al., 2022**
**134**	Subcore Sample	subcore_clayer	subcore consistency layer	The layer where sediment is consistently stratified by horizon, usually below the upper and inconsistent organic soil horizon/layer.				**Pawlowski et al., 2022**
**135**	Subcore Sample	subcore_notes	subcore notes	Subcore notes.				

Supplementary Table S4: Wet lab module fields: Expanded description of all fields (field, name), associated tables (table), field examples (example), related MIxS fields (MIxS), MIMARKS-SURVEY requirements (M-S), and field citations (citations) for the Wet Lab module.

id	table	field	name	description	example	MIxS v5	M-S	citations
**1**	Primer Pair	primer_set_name	primer set name	general name of the primer set.	mifishU			**Freeland, 2017**
**2**	Primer Pair	primer_target_gene	target gene	Targeted gene or locus name for marker gene studies (GSC Minimum Information about any Sequence; MIxS v5). This references the enumeration TargetGenes.	[12S, 16S, 18S, COI, ITS]	target_gene	M	**Bustin et al., 2009; Yilmaz et al., 2011; Miya et al., 2015; Collins et al., 2019**
**3**	Primer Pair	primer_subfragment	target subfragment	Name of subfragment of a gene or locus. Important to e.g. identify special regions on marker genes like V6 on 16S rRNA (GSC Minimum Information about any Sequence; MIxS v5). This references the enumeration SubFragments.	[V1, V2, V3, V4, V5, V6, V7, V8 V9, ITS1, ITS2, ITS3, ITS4]	target_subfragment	C	**Bustin et al., 2009; Yilmaz et al., 2011; Collins et al., 2019**
**4**	Primer Pair	primer_name_forward	name forward	PCR primers that were used to amplify the sequence of the targeted gene, locus or subfragment. This field should contain all the primers used for a single PCR reaction if multiple forward or reverse primers are present in a single PCR reaction. The primer sequence should be reported in uppercase letters (GSC Minimum Information about any Sequence; MIxS v5).	mifish_u_f	pcr_primers	C	Bustin et al., 2009; Yilmaz et al., 2011

id	table	field	name	description	example	MIxS v5	M-S	citations
**5**	Primer Pair	primer_name_reverse	name reverse	PCR primers that were used to amplify the sequence of the targeted gene, locus or subfragment. This field should contain all the primers used for a single PCR reaction if multiple forward or reverse primers are present in a single PCR reaction. The primer sequence should be reported in uppercase letters (GSC Minimum Information about any Sequence; MIxS v5).	mifish_u_r	pcr_primers	C	Bustin et al., 2009; Yilmaz et al., 2011
**6**	Primer Pair	primer_forward	forward	PCR primers that were used to amplify the sequence of the targeted gene, locus or subfragment. This field should contain all the primers used for a single PCR reaction if multiple forward or reverse primers are present in a single PCR reaction. The primer sequence should be reported in uppercase letters (GSC Minimum Information about any Sequence; MIxS v5).	GTCGGTAAAACTCGTGCCAGC	pcr_primers	C	Bustin et al., 2009; Yilmaz et al., 2011
**7**	Primer Pair	primer_reverse	reverse	PCR primers that were used to amplify the sequence of the targeted gene, locus or subfragment. This field should contain all the primers used for a single PCR reaction if multiple forward or reverse primers are present in a single PCR reaction. The primer sequence should be reported in uppercase letters (GSC Minimum Information about any Sequence; MIxS v5).	CATAGTGGGGTATCTAATCCCAGTTTG	pcr_primers	C	Bustin et al., 2009; Yilmaz et al., 2011

id	table	field	name	description	example	MIxS v5	M-S	citations
**8**	Primer Pair	primer_amplicon_length_min	min amplicon length	The estimated size of the genome prior to sequencing. Of particular importance in the sequencing of (eukaryotic) genome which could remain in draft form for a long or unspecified period (GSC Minimum Information about any Sequence; MIxS v5).	163	estimated_size	-	**Bustin et al., 2009; Yilmaz et al., 2011; Bylemans et al., 2018**
**9**	Primer Pair	primer_amplicon_length_max	max amplicon length	The estimated size of the genome prior to sequencing. Of particular importance in the sequencing of (eukaryotic) genome which could remain in draft form for a long or unspecified period (GSC Minimum Information about any Sequence; MIxS v5).	185	estimated_size	-	**Bustin et al., 2009; Yilmaz et al., 2011; Bylemans et al., 2018**
**10**	Primer Pair	primer_ref_biomaterial_url	ref biomaterial URL	Primary publication if isolated before genome publication; otherwise, primary genome report (GSC Minimum Information about any Sequence; MIxS v5).	https://doi.org/10.1098/rsos.150088	ref_biomaterial	-	**Yilmaz et al., 2011**
**11**	Primer Pair	primer_pair_notes	primer pair notes	Primer pair notes.				
**12**	Index Pair	index_i7	i7 Index	Molecular barcodes, called Multiplex Identifiers (MIDs), that are used to specifically tag unique samples in a sequencing run. Sequence should be reported in uppercase letters (GSC Minimum Information about any Sequence; MIxS v5).	GGACTCCT	mid	C	**Yilmaz et al., 2011**

id	table	field	name	description	example	MIxS v5	M-S	citations
**13**	Index Pair	i7_index_id	i7 Index ID	Molecular barcodes, called Multiplex Identifiers (MIDs), that are used to specifically tag unique samples in a sequencing run. Sequence should be reported in uppercase letters (GSC Minimum Information about any Sequence; MIxS v5).	A-N705	mid	C	**Yilmaz et al., 2011**
**14**	Index Pair	index_i5	i5 Index	Molecular barcodes, called Multiplex Identifiers (MIDs), that are used to specifically tag unique samples in a sequencing run. Sequence should be reported in uppercase letters (GSC Minimum Information about any Sequence; MIxS v5).	TTCTAGCT	mid	C	**Yilmaz et al., 2011**
**15**	Index Pair	i5_index_id	i5 Index ID	Molecular barcodes, called Multiplex Identifiers (MIDs), that are used to specifically tag unique samples in a sequencing run. Sequence should be reported in uppercase letters (GSC Minimum Information about any Sequence; MIxS v5).	C-S515	mid	C	**Yilmaz et al., 2011**
**16**	Index Pair	mixs_mid	MiXS mid format	PCR primers that were used to amplify the sequence of the targeted gene, locus or subfragment. This field is generated automatically on creation in the MIxS format i7_Index:{i7};I5_Index:{i5} (GSC Minimum Information about any Sequence; MIxS v5).	I7_Index:GGACTCCT;I5_Index:TTCTAGCT	mid	C	**Yilmaz et al., 2011**

id	table	field	name	description	example	MIxS v5	M-S	citations
**17**	Index Pair	index_adapter	adapter	Adapters provide priming sequences for both amplification and sequencing of the sample-library fragments. Both adapters should be reported; in uppercase letters (GSC Minimum Information about any Sequence; MIxS v5).	TCGTCGGCAGCGTCAGATGTGTATAAGCAG;GTCTCGTGGGCTCGGAGATGTGTATAAGAGACAG	adapters	C	**Yilmaz et al., 2011**
**18**	Index Removal Method	index_removal_method_name	index removal method name	general name of the index removal method.	beads			
**19**	Index Removal Method	index_removal_method_sop	index removal method SOP URL	Link to a literature reference, electronic resource or a standard operating procedure (SOP) (GSC Minimum Information about any Sequence; MIxS v5). This is a foreign key to the Standard Operating Procedure table.	https://www.index-removal-beads.com	sop	C	**Field et al., 2008; Bustin et al., 2009; Yilmaz et al., 2011; Bronner et al., 2009**
**20**	Size Selection Method	size_selection_method_name	size selection method name	general name of the size selection method	beads			
**21**	Size Selection Method	primer_set	primer set	The associated primer set used with this size selection method. This is a foreign key to the Primer Pair table.	mifishU			Bustin et al., 2009; Yilmaz et al., 2011
**22**	Size Selection Method	size_selection_sop	size selection method SOP URL	Link to a literature reference, electronic resource or a standard operating procedure (SOP) (GSC Minimum Information about any Sequence; MIxS v5). This is a foreign key to the Standard Operating Procedure table.	https://www.size-selection-beads.com, https://files.zymoresearch.com/protocols/_d4084_d4085_select-a-size_dna_clean_concentrator_magbead_kit.pdf	sop	C	Field et al., 2008; Bustin et al., 2009; Yilmaz et al., 2011
**23**	Quantification Method	quant_method_name	quantification method name	general name of the quantification method	[QuBit and qPCR, QuBit, qPCR, bioanalyzer, tape station, nanodrop, ...]			
**24**	Amplification Method	amplification_method_name	amplification method name	general name of the amplification method	[PCR, ...]	nucl_acid_amp	C	Field et al., 2008; Yilmaz et al., 2011

id	table	field	name	description	example	MIxS v5	M-S	citations
**25**	Amplification Method	amplification_sop	amplification method SOP URL	Nucleic acid amplification. Link to a literature reference, electronic resource or a standard operating procedure (SOP). Reference to amplification method; clean-up method (GSC Minimum Information about any Sequence; MIxS v5). This is a foreign key to the Standard Operating Procedure table.	https://www.pcr.com	sop, nucl_acid_amp	C, C	**Field et al., 2008; Yilmaz et al., 2011; Miya et al., 2015**
**26**	Extraction Method	extraction_method_name	extraction method name	general name of the extraction method	[Power Soil Pro, Power Water, PowerMax Soil, Blood and Tissue, ...]	nucl_acid_ext	C	**Field et al., 2008; Bustin et al., 2009; Yilmaz et al., 2011; Tsuji et al., 2019; Minamoto et al., 2021**
**27**	Extraction Method	extraction_method_manufacturer	extraction method manufacturer	manufacturer of the extraction method	[Qiagen, ...]	nucl_acid_ext	C	**Field et al., 2008; Bustin et al., 2009; Yilmaz et al., 2011; Minamoto et al., 2021**
**28**	Extraction Method	extraction_sop	extraction SOP URL	Nucleic acid extraction. Link to a literature reference, electronic resource or a standard operating procedure (SOP) (GSC Minimum Information about any Sequence; MIxS v5). This is a foreign key to the Standard Operating Procedure table.		nucl_acid_ext, sop	C, C	**Field et al., 2008; Bustin et al., 2009; Yilmaz et al., 2011; Minamoto et al., 2021**
**29**	Extraction	extraction_barcode	barcode	The associated barcode of the extraction. This is a foreign key to the Sample Barcode table.				**Minamoto et al., 2021**
**30**	Extraction	field_sample	field sample	The associated field sample of the extraction. This is a foreign key to the Field Sample table.				**Minamoto et al., 2021**
**31**	Extraction	extraction_control	extraction control	Booleain indication of whether the extraction is a control. This references the enumeration YesNo.	[yes, no]			

id	table	field	name	description	example	MIxS v5	M-S	citations
**32**	Extraction	extraction_control_type	extraction control type	If the extraction is a control, the type of control. A field control is deionized (DI) water exposed to air in the field. A lab control is a blank filter that was placed on the filter apparatus with fresh DI water (not the same DI water as the field control). An extraction control is a dry, unused filter that is put into a tube and extracted. A No Template Control is DNA free water used in PCR. A Mock Community is a positive control comprised of pre-selected specimens. An Environmental Standard is a positive control that is derived from a single water collection taken at a distinct location and time. This references the enumeration ControlTypes.	[field, lab, extraction, no_template_control, mock_community, env_standard, ...]			**Minamoto et al., 2021; Rees et al., 2014**
**33**	Extraction	process_location	process location	The location of the extraction. This is a foreign key to the Process Location table.				**Minamoto et al., 2021**
**34**	Extraction	extraction_datetime	extraction datetime	The date and time of the extraction.				**Minamoto et al., 2021**
**35**	Extraction	extraction_method	extraction method	The associated extraction method. This is a foreign key to the Extraction Method table.		nucl_acid_ext	C	**Field et al., 2008; Bustin et al., 2009; Yilmaz et al., 2011; Minamoto et al., 2021**
**36**	Extraction	extraction_first_name	first name	The first name of the personnel responsible for the extraction.				**Minamoto et al., 2021**
**37**	Extraction	extraction_last_name	last name	The last name of the personnel responsible for the extraction.				**Minamoto et al., 2021**
**38**	Extraction	extraction_volume	extraction volume	The final volume of the extraction.				**Bustin et al., 2009; Minamoto et al., 2021**
**39**	Extraction	extraction_volume_units	extraction volume units	The units of the final volume of the extraction. This references the enumeration VolUnits.	[microliter, milliliter, ...]			**Bustin et al., 2009; Minamoto et al., 2021**
**40**	Extraction	quantification_method	quantification method	The quantification method. This is a foreign key to the Quantification Method table.				**Minamoto et al., 2021**
**41**	Extraction	extraction_concentration	extraction concentration	The final concentration of the extraction.				**Bustin et al., 2009; Minamoto et al., 2021**

id	table	field	name	description	example	MIxS v5	M-S	citations
**42**	Extraction	extraction_concentration_units	extraction concentration units	The units of the final concentration of the extraction. This references the enumeration ConcentrationUnits.	[nanograms_per_microliter, nanograms_per_milliliter, picograms_per_microliter, nanomolar, picomolar, ...]			**Bustin et al., 2009; Minamoto et al., 2021**
**43**	Extraction	extraction_notes	extraction notes	Extraction notes.				
**44**	PCR Replicate	pcr_replicate_results	results	The results of the PCR replicate.				**Bustin et al., 2009**
**45**	PCR Replicate	pcr_replicate_results_units	results units	The results units. This references the enumeration PcrUnits.	[quantification cycle (Cq; qPCR), copy number (cp; ddPCR), copies per microliter (copy/µL; ddPCR)]			**Bustin et al., 2009**
**46**	PCR Replicate	pcr_replicate_notes	PCR replicate notes	PCR replicate notes.				
**47**	PCR	pcr_experiment_name	PCR experiment name	The experiment name of the PCR.				**Miya et al., 2015**
**48**	PCR	pcr_type	PCR type	The type of the PCR. This references the enumeration PcrTypes.	[ddPCR, qPCR]			**Miya et al., 2015**
**49**	PCR	pcr_datetime	PCR datetime	The date and time of the PCR.				**Miya et al., 2015**
**50**	PCR	process_location	process location	The location of the extraction. This is a foreign key to the Process Location table.				**Miya et al., 2015**
**51**	PCR	extraction	extraction	The associated extraction used in PCR. This is a foreign key to the Extraction table.				**Bustin et al., 2009**
**52**	PCR	primer_set	primer set	The associated primer set used in the PCR. This is a foreign key to the Primer Pair table.				**Bustin et al., 2009; Miya et al., 2015; Collins et al., 2019**
**53**	PCR	pcr_first_name	first name	The first name of the personnel responsible for running PCR				
**54**	PCR	pcr_last_name	last name	The last name of the personnel responsible for running PCR				
**55**	PCR	pcr_probe	probe	The probe used in the PCR.				**Bustin et al., 2009; Miya et al., 2015**
**56**	PCR	pcr_results	results	The results of the PCR				**Bustin et al., 2009; Miya et al., 2015; Collins et al., 2019**

id	table	field	name	description	example	MIxS v5	M-S	citations
**57**	PCR	pcr_results_units	results units	The results units. This references the enumeration PcrUnits.	[quantification cycle (Cq; qPCR), copy number (cp; ddPCR), copies per microliter (copy/µL; ddPCR)]			**Bustin et al., 2009; Miya et al., 2015; Collins et al., 2019**
**58**	PCR	pcr_replicate	PCR replicate	Any replicates associated with the PCR. This is a foreign key to the PCR Replicate table.				**Bustin et al., 2009; Miya et al., 2015; Collins et al., 2019**
**59**	PCR	pcr_thermal_cond	PCR thermal conditions	Description of reaction conditions and components for PCR in the form of: "initial denaturation:degrees_minutes; annealing:degrees_minutes; elongation: degrees_minutes; final elongation:degrees_minutes; total cycles" (GSC Minimum Information about any Sequence; MIxS v5).	initial denaturation:94degC_1.5min; annealing=...	pcr_cond	C	**Field et al., 2008; Bustin et al., 2009; Yilmaz et al., 2011; Miya et al., 2015; Collins et al., 2019**
**60**	PCR	pcr_sop	PCR SOP URL	Link to a literature reference, electronic resource or a standard operating procedure (SOP) (GSC Minimum Information about any Sequence; MIxS v5). This is a foreign key to the Standard Operating Procedure table.		sop	C	**Bustin et al., 2009; Miya et al., 2015**
**61**	PCR	pcr_notes	PCR notes	PCR notes				
**62**	Library Prep	lib_prep_experiment_name	library prep experiment name	The experiment name of the library prep.				
**63**	Library Prep	lib_prep_datetime	library prep datetime	The date and time of the library prep.				
**64**	Library Prep	process_location	process location	The location of the library prep. This is a foreign key to the Process Location table.				
**65**	Library Prep	extraction	extraction	Nucleic acid extraction. Foreign key to extraction table.		nucl_acid_ext	C	**Yilmaz et al., 2011**

id	table	field	name	description	example	MIxS v5	M-S	citations
**66**	Library Prep	amplification_method_name	amplification method	The associated amplification method of the library prep. This is a foreign key to the Amplification Method table.		nucl_acid_amp, sop	C, C	Field et al., 2008; Bustin et al., 2009; Yilmaz et al., 2011
**67**	Library Prep	primer_set	primer set	The associated primer set of the library prep. This is a foreign key to the Primer Set table.				
**68**	Library Prep	size_selection_method_name	size selection method	Each associated size selection method of the library prep. This is a foreign key to the Size Selection Method table.				**Bronner et al., 2009**
**69**	Library Prep	index_pair	index pair	Each associated index pair of the library prep. This is a foreign key to the Index Pair table.				**Bronner et al., 2009**
**70**	Library Prep	index_removal_method_name	index removal method	Each associated index removal method of the library prep. This is a foreign key to the Index Removal Method table.				**Bronner et al., 2009**
**71**	Library Prep	quantification_method	quantification method	The associated quantification method of the library prep. This is a foreign key to the Quantification Method table.				**Bronner et al., 2009**
**72**	Library Prep	lib_prep_final_concentration	final concentration	The final concentration of the library prep				**Bronner et al., 2009**
**73**	Library Prep	lib_prep_final_concentration_units	final concentration units	The final concentration units of the library prep. This references the enumeration ConcentrationUnits.	[nanograms_per_microliter, nanograms_per_milliliter, picograms_per_microliter, nanomolar, picomolar, ...]			**Bronner et al., 2009**

id	table	field	name	description	example	MIxS v5	M-S	citations
**74**	Library Prep	lib_prep_kit	library prep kit	The library prep kit of the library prep. This references the enumeration LibPrepKits.	[IDT-ILMN TruSeq DNA-RNA UD 24 indexes, IDT-ILMN TruSeq DNA-RNA UD 96 indexes, Nextera DNA, Nextera DNA CD INdexes- 24 indexes, Nextera DNA CD INdexes- 96 indexes, Nextera Mate Pair, Nextera Rapid Capture Enrichment, Nextera XT, Nextera XT V2, ScriptSeq Complete, ScriptSeq V2, SureCell Single Cell RNA 1.0, SureCell WTA 3', TruSeq Amplicon, TruSeq DNA Methylation, TruSeq DNA-RNA CD Indexes 96 Indexes, TruSeq DNA-RNA Single Indexes Set A&B, TruSeq Methyl Capture EPIC, TruSeq Ribo Profil, TruSeq Small RNA, TruSeq Targeted RNA Expression, TruSight Amplicon Panels, TruSight Enrichment Panels, TruSight RNA Fusion, TruSight Tumor 15, TruSight Tumor 126, AmpliSeq Library PLUS for Illumina (96), Custom]			**Bronner et al., 2009**
**75**	Library Prep	lib_prep_type	library prep type	The library prep kit type. This references the enumeration LibPrepType.	[Amplicon Sequencing, 16s rRNA Sequencing, Shotgun Sequencing, Whole-Genome Sequencing, De Novo Sequencing]			

id	table	field	name	description	example	MIxS v5	M-S	citations
**76**	Library Prep	lib_prep_layout	library layout	Specify whether to expect single-end, paired-end, or other configuration of reads (GSC Minimum Information about any Sequence; MIxS v5). This references the enumeration LibLayouts.	[paired-end, single-end, vector, other, ...]	lib_const_meth (v4), lib_layout (v5)	C	Field et al., 2008; Yilmaz et al., 2011
**77**	Library Prep	lib_prep_thermal_cond	library prep thermal conditions	Description of reaction conditions and components for PCR in the form of: "initial denaturation:degrees_minutes; annealing:degrees_minutes; elongation: degrees_minutes; final elongation:degrees_minutes; total cycles" (GSC Minimum Information about any Sequence; MIxS v5).	initial denaturation:94degC_1.5min; annealing: ...	pcr_cond, sop	C, C	Field et al., 2008; Bustin et al., 2009; Yilmaz et al., 2011
**78**	Library Prep	lib_prep_sop	library prep SOP URL	Link to a literature reference, electronic resource or a standard operating procedure (SOP) (GSC Minimum Information about any Sequence; MIxS v5). This is a foreign key to the Standard Operating Procedure table.		sop	C	Field et al., 2008; Bustin et al., 2009; Yilmaz et al., 2011
**79**	Library Prep	lib_prep_notes	library prep notes	Library prep notes.				
**80**	Pooled Library	pooled_lib_label	pooled library label	The label of the pooled library				
**81**	Pooled Library	pooled_lib_datetime	pooled library datetime	The date and time the library was pooled				
**82**	Pooled Library	pooled_lib_barcode	pooled library barcode	The barcode of the pooled library. This is a foreign key to the Sample Barcode table.				
**83**	Pooled Library	process_location	process location	The location the library was pooled. This is a foreign key to the Process Location table.				

id	table	field	name	description	example	MIxS v5	M-S	citations
**84**	Pooled Library	library_prep	library prep	Each associated library prep of the pooled library. This is a foreign key to the Library Prep table.				**Bronner et al., 2009**
**85**	Pooled Library	quantification_method	quantification method	The associated quantification method of the pooled library. This is a foreign key to the Quantification Method table.				**Bronner et al., 2009**
**86**	Pooled Library	pooled_lib_concentration	pooled library concentration	The final concentration of the pooled library.				**Bronner et al., 2009**
**87**	Pooled Library	pooled_lib_concentration_units	pooled library concentration units	The final concentration units of the pooled library. This references the enumeration ConcentrationUnits.	[nanograms_per_microliter, nanograms_per_milliliter, picograms_per_microliter, nanomolar, picomolar, ...]			**Bronner et al., 2009**
**88**	Pooled Library	pooled_lib_volume	pooled library volume	The final volume of the pooled library.				
**89**	Pooled Library	pooled_lib_volume_units	pooled library volume units	The units of the final volume of the pooled library. This references the enumeration VolUnits.	[microliter, milliliter, ...]			
**90**	Pooled Library	pooled_lib_notes	pooled library notes	Pooled library notes.				
**91**	Run Prep	run_prep_label	run prep label	The label of the run prep.				
**92**	Run Prep	run_prep_datetime	run prep datetime	The date and time of run prep.				
**93**	Run Prep	process_location	process location	The associated location of run prep. This is a foreign key to the Process Location table.				
**94**	Run Prep	pooled_library	pooled library	Each associated pooled library of the run prep. This is a foreign key to the Pooled Library table.				
**95**	Run Prep	quantification_method	quantification method	The quantification method. This is a foreign key to the Quantification Method table.				**Bronner et al., 2009**
**96**	Run Prep	run_prep_concentration	run prep concentration (pre PhiX)	The final library concentration prior to adding in PhiX.				

id	table	field	name	description	example	MIxS v5	M-S	citations
**97**	Run Prep	run_prep_concentration_units	run prep concentration units (pre PhiX)	The final library concentration units. This references the enumeration ConcentrationUnits.	[nanograms_per_microliter, nanograms_per_milliliter, picograms_per_microliter, nanomolar, picomolar, ...]			
**98**	Run Prep	run_prep_phix_spike_in	PhiX spike in	The PhiX spike-in of the run prep.				
**99**	Run Prep	run_prep_phix_spike_in_units	PhiX spike in units	The PhiX spike-in units of the run prep. This references the enumeration PhiXConcentrationUnits.	[picomolar (pM), Percent (%), ...]			
**100**	Run Prep	run_prep_notes	run prep notes	Run prep notes.				
**101**	Run Result	run_experiment_name	run experiment name	The experiment name of the run result. Typically reported in Illumina output SampleSheet.csv				
**102**	Run Result	run_id	run ID	The run ID of the run result. Typically reported in Illumina output RunInfo.xml	220409_M04167_0008_000000000-K7WKT			
**103**	Run Result	run_date	run date	The run date of the run result. Typically reported in Illumina output RunInfo.xml				
**104**	Run Result	process_location	process location	The associated location of the run result. This is a foreign key to the Process Location table.				
**105**	Run Result	run_prep	run prep	The associated run prep of the run result. This is a foreign key to the Run Prep table.				
**106**	Run Result	run_completion_datetime	run completion datetime	The run completion date and time. Typically reported in Illumina output CompletedJobInfo.xml				
**107**	Run Result	run_instrument	run instrument	The run instrument of the run result. Typically reported in Illumina output RunInfo.xml	M04040			
**108**	FASTQ File	uuid	uuid	A universally unique identifier for each FASTQ file.	cf4494e1-c7b8-4abf-ba6e-07e4fe8608b1			

id	table	field	name	description	example	MIxS v5	M-S	citations
**109**	FASTQ File	run_result	run result	The associated run result of the FASTQ file. This is a foreign key to the Run Result table.				
**110**	FASTQ File	extraction	extraction	The associated extraction of the FASTQ file. This is a foreign key to the Extraction table.				
**111**	FASTQ File	primer_set	primer set	The associated primer set of the FASTQ file. This is a foreign key to the Primer Pair table.				
**112**	FASTQ File	fastq_datafile	FASTQ datafile	The FASTQ datafile.	eLP_O01_22w_0001_S38_L001_R1_001.fastq.gz			
**113**	FASTQ File	submitted_to_insdc	submitted to insdc	Depending on the study (large-scale e.g. done with next generation sequencing technology, or small-scale) sequences have to be submitted to SRA (Sequence Read Archive), DRA (DDBJ Read Archive) or via the classical Webin/Sequin systems to Genbank, ENA and DDBJ. Although this field is mandatory, it is meant as a self-test field, therefore it is not necessary to include this field in contextual data submitted to databases (GSC Minimum Information about any Sequence; MIxS v5). This references the enumeration YesNo.	[yes, no]	submitted_to_insdc	M	Field et al., 2008; Yilmaz et al., 2011
**114**	FASTQ File	insdc_url	insdc URL	If the FASTQ file was submitted to insdc, the URL to the associated repository.	https://www.ncbi.nlm.nih.gov/sra/SRX5301973			

id	table	field	name	description	example	MIxS v5	M-S	citations
**115**	FASTQ File	seq_meth	sequencing method	Sequencing method used. This references the enumeration SeqMethods.	[MinION, GridION, PromethION, 454 GS, 454 GS 20, 454 GS FLX, 454 GS FLX+, 454 GS FLX Titanium, 454 GS Junior, Illumina Genome Analyzer, Illumina Genome Analyzer II, Illumina Genome Analyzer IIx, Illumina HiSeq 4000, Illumina HiSeq 3000, Illumina HiSeq 2500, Illumina HiSeq 2000, Illumina HiSeq 1500, Illumina HiSeq 1000, Illumina HiScanSQ, Illumina MiSeq, Illumina HiSeq X Five, Illumina HiSeq X Ten, Illumina NextSeq 500, Illumina NextSeq 550, AB SOLiD System, AB SOLiD System 2.0, AB SOLiD System 3.0, AB SOLiD 3 Plus System, AB SOLiD 4 System, AB SOLiD 4hq System, AB SOLiD PI System, AB 5500 Genetic Analyzer, AB 5500xl Genetic Analyzer, AB 5500xl-W Genetic Analysis System, Ion Torrent PGM, Ion Torrent Proton, Ion Torrent S5, Ion Torrent S5 XL, PacBio RS, PacBio RS II, Sequel, AB 3730xL Genetic Analyzer, AB 3730 Genetic Analyzer, AB 3500xL Genetic Analyzer, AB 3500 Genetic Analyzer, AB 3130xL Genetic Analyzer, AB 3130 Genetic Analyzer, AB 310 Genetic Analyzer, BGISEQ-500]	seq_meth	M	Field et al., 2008; Yilmaz et al., 2011

id	table	field	name	description	example	MIxS v5	M-S	citations
**116**	FASTQ File	investigation_type	investigation type	Nucleic Acid Sequence Report is the root element of all MIGS/MIMS compliant reports as standardized by Genomic Standards Consortium. This field is either eukaryote,bacteria,virus,plasmid,organelle, metagenome,mimarks-survey, mimarks-specimen, metatranscriptome, single amplified genome, metagenome-assembled genome, or uncultivated viral genome (GSC Minimum Information about any Sequence; MIxS v5). This references the enumeration InvestigationTypes.	[eukaryote, bacteria archaea, plasmid, virus, organelle, metagenome,mimarks-survey, mimarks-specimen, metatranscriptome, single amplified genome, metagenome-assembled genome, or uncultivated viral genomes]	investigation_type	M	Field et al., 2008; Yilmaz et al., 2011

Supplementary Table S5: Bioinformatics module fields: Expanded description of all fields (field, name), associated tables (table), field examples (example), related MIxS fields (MIxS), MIMARKS-SURVEY requirements (M-S), and field citations (citations) for the Bioinformatics module.

id	table	field	name	description	example	MIxS	M-S	citations
**1**	Quality Metadata	fastq_file	FASTQ file	The associated FASTQ file. This is a foreign key to the FASTQ file table.				
**2**	Quality Metadata	process_location	process location	The location of the taxonomic annotation. This is a foreign key to the Process Location table.	[Texas Advanced Computing Center (TACC), Bigelow, ...]			
**3**	Quality Metadata	analysis_label	analysis label	The label of the analysis.				
**4**	Quality Metadata	analysis_datetime	analysis datetime	The date and time of the analysis.				
**5**	Quality Metadata	analyst_first_name	analyst first name	The first name of personnel running the analysis.				
**6**	Quality Metadata	analyst_last_name	analyst last name	The last name of the personnel running the analysis.				
**7**	Quality Metadata	seq_quality_check	sequence quality check	Indicate if the sequence has been called by automatic systems (none) or undergone a manual editing procedure (e.g. by inspecting the raw data or chromatograms). Applied only for sequences that are not submitted to SRA,ENA or DRA (GSC Minimum Information about any Sequence; MIxS v5). This references the enumeration QualityChecks.	[none, manually edited]	seq_qualitycheck (v4) seq_quality_check (v5)	C	**Yilmaz et al., 2011**
**8**	Quality Metadata	trim_length_forward	trim length forward	the length to trim the forward reads in base pairs (bp)	250	seq_qualitycheck (v4) seq_quality_check (v5)	C	**Yilmaz et al., 2011**
**9**	Quality Metadata	trim_length_reverse	trim length reverse	the length to trim the reverse reads in base pairs (bp)	200	seq_qualitycheck (v4) seq_quality_check (v5)	C	**Yilmaz et al., 2011**
**10**	Quality Metadata	min_read_length	minimum read length	the minimum read length filtered in base pairs (bp)	150	seq_qualitycheck (v4) seq_quality_check (v5)	C	**Yilmaz et al., 2011**

id	table	field	name	description	example	MIxS	M-S	citations
**11**	Quality Metadata	max_read_length	maximum read length	the maximum read length filtered in base pairs (bp)	400	seq_qualitycheck (v4) seq_quality_check (v5)	C	**Yilmaz et al., 2011**
**12**	Quality Metadata	analysis_sop	analysis SOP url	Link to a literature reference, electronic resource or a standard operating procedure (SOP) (GSC Minimum Information about any Sequence; MIxS v5). This is a foreign key to the Standard Operating Procedure table.		sop, url	C, C	**Field et al., 2008**
**13**	Quality Metadata	analysis_script_repo_url	analysis script repo url	Link to the repository script used to run the analysis.		sop, url	C, C	**Field et al., 2008**
**14**	Denoise Cluster Method	denoise_cluster_method_name	method name	name of denoising or clustering method	[DADA2, QIIME2-Deblur]	votu_class_appr	-	Yilmaz et al., 2011; Callahan et al., 2016; Amir et al., 2017; Bolyen et al., 2019
**15**	Denoise Cluster Method	denoise_cluster_method_software_package	method software package	name of software package	[QIIME2, MOTHUR]	votu_class_appr	-	Yilmaz et al., 2011; Bolyen et al., 2019; Schloss et al., 2009
**16**	Denoise Cluster Method	denoise_cluster_method_env_url	environment file URL	list of installed packages in environment used to run bioinformatics pipeline	https://github.com/Maine-eDNA/medna-metadata/blob/main/requirements/base.txt	sop, url	C, C	**Yilmaz et al., 2011**
**17**	Denoise Cluster Metadata	quality_metadata	quality metadata	The associated quality metadata. This is a foreign key to the Quality Metadata table.		seq_qualitycheck (v4) seq_quality_check (v5)	C	**Yilmaz et al., 2011**
**18**	Denoise Cluster Metadata	process_location	process location	The location of the denoising or clustering. This is a foreign key to the Process Location table.	[Texas Advanced Computing Center (TACC), Bigelow, ...]			
**19**	Denoise Cluster Metadata	analysis_label	analysis label	The label of the analysis.				
**20**	Denoise Cluster Metadata	analysis_datetime	analysis datetime	Date and time of the analysis.				
**21**	Denoise Cluster Metadata	analyst_first_name	analyst first name	The first name of personnel running the analysis.				
**22**	Denoise Cluster Metadata	analyst_last_name	analyst last name	The last name of the personnel running the analysis.				

id	table	field	name	description	example	MIxS	M-S	citations
**23**	Denoise Cluster Metadata	denoise_cluster_method	denoise cluster method	The associated denoise or cluster method. This is a foreign key to the Denoise Cluster Method table.		votu_class_appr	-	Yilmaz et al., 2011; Callahan et al., 2016; Amir et al., 2017; Bolyen et al., 2019
**24**	Denoise Cluster Metadata	chimera_check	chimera check	A chimeric sequence, or chimera for short, is a sequence comprised of two or more phylogenetically distinct parent sequences. Chimeras are usually PCR artifacts thought to occur when a prematurely terminated amplicon reanneals to a foreign DNA strand and is copied to completion in the following PCR cycles. The point at which the chimeric sequence changes from one parent to the next is called the breakpoint or conversion point (GSC Minimum Information about any Sequence; MIxS v5).	name and version of software	chimera_check	C	**Yilmaz et al., 2011**
**25**	Denoise Cluster Metadata	analysis_sop	analysis SOP url	Link to a literature reference, electronic resource or a standard operating procedure (SOP) (GSC Minimum Information about any Sequence; MIxS v5). This is a foreign key to the Standard Operating Procedure table.		sop, url	C, C	**Field et al., 2008**
**26**	Denoise Cluster Metadata	analysis_script_repo_url	analysis script repo url	Link to the repository script used to run the analysis.		sop, url	C, C	**Field et al., 2008**
**27**	Feature Output	denoise_cluster_metadata	denoise cluster metadata	The associated denoise cluster metadata. This is a foreign key to the Denoise Cluster Metadata table.		votu_class_appr	-	**Yilmaz et al., 2011**
**28**	Feature Output	feature_id	feature id	The feature id.				
**29**	Feature Output	feature_sequence	feature sequence	The feature sequence.				

id	table	field	name	description	example	MIxS	M-S	citations
**30**	Feature Read	feature	feature	The associated feature. This is a foreign key to the Feature Output table.				
**31**	Feature Read	extraction	extraction	The associated extraction. This is a foreign key to the Extraction table.		nucl_acid_ext	C	Field et al., 2008; Bustin et al., 2009; Yilmaz et al., 2011
**32**	Feature Read	number_reads	number reads	The total number of reads of the feature.				
**33**	Reference Database	refdb_name	reference database name	The reference database name.		ref_db	-	Yilmaz et al., 2011; Yilmaz et al., 2014; Guillou et al., 2012; Iwasaki et al., 2013; Sato et al., 2018; Bucklin et al., 2021
**34**	Reference Database	refdb_version	reference database version	The version of the reference database.		ref_db	-	Yilmaz et al., 2011; Yilmaz et al., 2014; Guillou et al., 2012; Iwasaki et al., 2013; Sato et al., 2018; Bucklin et al., 2021
**35**	Reference Database	refdb_datetime	reference database datetime	The date and time the version of the reference database was created.		ref_db	-	Yilmaz et al., 2011; Yilmaz et al., 2014; Guillou et al., 2012; Iwasaki et al., 2013; Sato et al., 2018; Bucklin et al., 2021
**36**	Reference Database	redfb_coverage_sco re	coverage score	The taxonomic coverage score of the reference database as a percentage.		ref_db	-	Yilmaz et al., 2011; Yilmaz et al., 2014; Guillou et al., 2012; Iwasaki et al., 2013; Sato et al., 2018; Bucklin et al., 2021
**37**	Reference Database	refdb_repo_url	reference database url	The reference database URL.		ref_db, url	-, C	Yilmaz et al., 2011; Yilmaz et al., 2014; Guillou et al., 2012; Iwasaki et al., 2013; Sato et al., 2018; Bucklin et al., 2021
**38**	Reference Database	refdb_notes	reference database notes	Reference database notes.				
**39**	Taxon Domain	taxon_domain	domain	list of domains.		tax_class	-	**Yilmaz et al., 2011**
**40**	Taxon Domain	taxon_url	domain URL	if applicable, URL to domain.		tax_class, url	-, C	**Yilmaz et al., 2011**
**41**	Taxon Kingdom	taxon_kingdom	kingdom	list of kingdoms with associated domains.		tax_class	-	**Yilmaz et al., 2011**

id	table	field	name	description	example	MIxS	M-S	citations
**42**	Taxon Kingdom	taxon_domain	domain	The related domain. This is a foreign key to the Taxon Domain table.				
**43**	Taxon Kingdom	taxon_url	kingdom URL	if applicable, URL to kingdom		tax_class, url	-, C	**Yilmaz et al., 2011**
**44**	Taxon Supergroup	taxon_supergroup	supergroup	list of supergroups with associated domains.		tax_class	-	**Yilmaz et al., 2011**
**45**	Taxon Supergroup	taxon_kingdom	kingdom	The related kingdom. This is a foreign key to the Taxon Kingdom table.				
**46**	Taxon Supergroup	taxon_url	supergroup URL	if applicable, URL to the supergroup.		tax_class, url	-, C	**Yilmaz et al., 2011**
**47**	Taxon Phylum/Division	taxon_phylum_division	phylum	list of phylums/divisions with associated kingdoms and domains.		tax_class	-	**Yilmaz et al., 2011**
**48**	Taxon Phylum/Division	taxon_supergroup	supergroup	The related supergroup. This is a foreign key to the Taxon Supergroup table.				
**49**	Taxon Phylum/Division	taxon_url	phylum URL	if applicable, URL to phylum.		tax_class, url	-, C	**Yilmaz et al., 2011**
**50**	Taxon Class	taxon_class	class	list of classes with associated phylums, kingdoms, and domains.		tax_class	-	**Yilmaz et al., 2011**
**51**	Taxon Class	taxon_phylum_division	phylum/division	The related phylum/division. This is a foreign key to the Taxon Phylum Division table.				
**52**	Taxon Class	taxon_url	class URL	if applicable, URL to class		tax_class, url	-, C	**Yilmaz et al., 2011**
**53**	Taxon Order	taxon_order	order	list of orders with associated classes, phylums, kingdoms, and domains		tax_class	-	**Yilmaz et al., 2011**
**54**	Taxon Order	taxon_class	class	The related class. This is a foreign key to the Taxon Class table.				
**55**	Taxon Order	taxon_url	order URL	if applicable, URL to order.		tax_class, url	-, C	**Yilmaz et al., 2011**
**56**	Taxon Family	taxon_family	family	list of families with associated orders, classes, phylums, kingdoms, and domains.		tax_class	-	**Yilmaz et al., 2011**

id	table	field	name	description	example	MIxS	M-S	citations
**57**	Taxon Family	taxon_order	order	The related order. This is a foreign key to the Taxon Order table.				
**58**	Taxon Family	taxon_url	family URL	if applicable, URL to family.		tax_class, url	-, C	**Yilmaz et al., 2011**
**59**	Taxon Genus	taxon_genus	genus	list of genuses with associated families, orders, classes, phylums, kingdoms, and domains.		tax_class	-	**Yilmaz et al., 2011**
**60**	Taxon Genus	taxon_family	family	The related family. This is a foreign key to the Taxon Family table.				
**61**	Taxon Genus	taxon_url	genus URL	if applicable, URL to genus.		tax_class, url	-, C	**Yilmaz et al., 2011**
**62**	Taxon Species	taxon_species	species	list of species with associated genuses, families, orders, classes, phylums, kingdoms, and domains.		tax_class	-	**Yilmaz et al., 2011**
**63**	Taxon Species	taxon_common_name	common name	list of common names with associated species, genus, families, orders, classes, phylums, kingdoms, and domains	Alewife	tax_class	-	**Yilmaz et al., 2011**
**64**	Taxon Species	is_endemic	endemic	Booleain indication of whether the species is endemic to the study region. This references the enumeration YesNo.	[yes, no]			
**65**	Taxon Species	taxon_genus	genus	The related genus. This is a foreign key to the Taxon Genus table.				
**66**	Taxon Species	taxon_url	species URL	if applicable, URL to species.		tax_class, url	-, C	**Yilmaz et al., 2011**
**67**	Annotation Method	annotation_method_name	annotation method name	the name of the taxonomic annotation method	[BLAST+, Multinomial Naive Bayes (Feature Classifier)]	sim_search_meth, tax_class	-, -	Yilmaz et al., 2011; Altschul et al., 1990; Camacho et al., 2009; Bokulich et al., 2018
**68**	Annotation Method	annotation_method_software_package	method software package	name of software package	[QIIME2, MOTHUR, BLAST]	sim_search_meth, tax_class	-, -	**Yilmaz et al., 2011; Bolyen et al., 2019**

id	table	field	name	description	example	MIxS	M-S	citations
**69**	Annotation Method	annotation_method_env_url	environment file URL	list of installed packages in environment used to run bioinformatics pipeline	https://github.com/Maine-eDNA/medna-metadata/blob/main/requirements/base.txt	sop, url, sim_search_meth, tax_class	C, C, -, -	**Yilmaz et al., 2011**
**70**	Annotation Metadata	analysis_label	analysis label	The label of the analysis.				
**71**	Annotation Metadata	process_location	process location	The location of the taxonomic annotation. This is a foreign key to the Process Location table.	[Texas Advanced Computing Center (TACC), Bigelow, ...]			
**72**	Annotation Metadata	denoise_cluster_metadata	denoise cluster metadata	The associated denoise cluster metadata. This is a foreign key to the Denoise Cluster Metadata table.		votu_class_appr	-	**Yilmaz et al., 2011**
**73**	Annotation Metadata	analysis_datetime	analysis datetime	The date and time of the analysis.				
**74**	Annotation Metadata	annotation_method	annotation method	The associated taxonomic annotation method. This is a foreign key to the Annotation Method table.		sim_search_meth, tax_class	-, -	Yilmaz et al., 2011; Altschul et al., 1990; Camacho et al., 2009; Bokulich et al., 2018
**75**	Annotation Metadata	analyst_first_name	analyst first name	The first name of personnel running the analysis.				
**76**	Annotation Metadata	analyst_last_name	analyst last name	The last name of personnel running the analysis.				
**77**	Annotation Metadata	analysis_sop	analysis SOP URL	Link to a literature reference, electronic resource or a standard operating procedure (SOP) (GSC Minimum Information about any Sequence; MIxS v5). This is a foreign key to the Standard Operating Procedure table.		sop, url	C, C	**Field et al., 2008**
**78**	Annotation Metadata	analysis_script_repo_url	analysis script repo URL	Link to the repository script used to run the analysis.		sop, url	C, C	**Field et al., 2008**
**79**	Taxonomic Annotation	feature	feature	The associated feature. This is a foreign key to the Feature Output table.				

id	table	field	name	description	example	MIxS	M-S	citations
**80**	Taxonomic Annotation	annotation_metadata	annotation metadata	The associated annotation metadata. This is a foreign key to the Annotation Metadata table.		sim_search_meth, tax_class	-, -	**Yilmaz et al., 2011; Balvočiūtė and Huson, 2017; Beiko et al., 2015**
**81**	Taxonomic Annotation	reference_database	reference database	The associated reference database. This is a foreign key to the Reference Database table.	[Silva, PR2, unite, rdp ...]	ref_db	-	**Yilmaz et al., 2011; Balvočiūtė and Huson, 2017; Beiko et al., 2015**
**82**	Taxonomic Annotation	confidence	confidence	The confidence score of the assigned taxonomy.		sim_search_meth, tax_class	-, -	**Yilmaz et al., 2011; Balvočiūtė and Huson, 2017; Beiko et al., 2015**
**83**	Taxonomic Annotation	ta_taxon	taxon	The taxon annotated by the annotation method.		sim_search_meth, tax_class	-, -	**Yilmaz et al., 2011; Balvočiūtė and Huson, 2017; Beiko et al., 2015**
**84**	Taxonomic Annotation	ta_domain	domain	the domain annotated by the annotation method.		sim_search_meth, tax_class	-, -	**Yilmaz et al., 2011; Balvočiūtė and Huson, 2017; Beiko et al., 2015**
**85**	Taxonomic Annotation	ta_kingdom	kingdom	the kingdom annotated by the annotation method.		sim_search_meth, tax_class	-, -	**Yilmaz et al., 2011; Balvočiūtė and Huson, 2017; Beiko et al., 2015**
**86**	Taxonomic Annotation	ta_supergroup	supergroup	the supergroup annotated by the annotation method.		sim_search_meth, tax_class	-, -	**Yilmaz et al., 2011; Balvočiūtė and Huson, 2017; Beiko et al., 2015**
**87**	Taxonomic Annotation	ta_phylum_division	phylum/division	the phylum or division annotated by the annotation method.		sim_search_meth, tax_class	-, -	**Yilmaz et al., 2011; Balvočiūtė and Huson, 2017; Beiko et al., 2015**
**88**	Taxonomic Annotation	ta_class	class	the class annotated by the annotation method.		sim_search_meth, tax_class	-, -	**Yilmaz et al., 2011; Balvočiūtė and Huson, 2017; Beiko et al., 2015**
**89**	Taxonomic Annotation	ta_order	order	the order annotated by the annotation method.		sim_search_meth, tax_class	-, -	**Yilmaz et al., 2011; Balvočiūtė and Huson, 2017; Beiko et al., 2015**
**90**	Taxonomic Annotation	ta_family	family	the family annotated by the annotation method.		sim_search_meth, tax_class	-, -	**Yilmaz et al., 2011; Balvočiūtė and Huson, 2017; Beiko et al., 2015**
**91**	Taxonomic Annotation	ta_genus	genus	the genus annotated by the annotation method.		sim_search_meth, tax_class	-, -	**Yilmaz et al., 2011; Balvočiūtė and Huson, 2017; Beiko et al., 2015**

id	table	field	name	description	example	MIxS	M-S	citations
**92**	Taxonomic Annotation	ta_species	species	the species annotated by the annotation method.		sim_search_meth, tax_class	-, -	**Yilmaz et al., 2011; Balvočiūtė and Huson, 2017; Beiko et al., 2015**
**93**	Taxonomic Annotation	ta_common_name	common name	the common name of the species annotated by the annotation method.		sim_search_meth, tax_class	-, -	**Yilmaz et al., 2011; Balvočiūtė and Huson, 2017; Beiko et al., 2015**
**94**	Taxonomic Annotation	manual_domain	manual domain	Manually assigned domain.		tax_class	-	**Yilmaz et al., 2011; Balvočiūtė and Huson, 2017; Beiko et al., 2015**
**95**	Taxonomic Annotation	manual_kingdom	manual kingdom	Manually assigned kingdom.		tax_class	-	**Yilmaz et al., 2011; Balvočiūtė and Huson, 2017; Beiko et al., 2015**
**96**	Taxonomic Annotation	manual_supergroup	manual supergroup	Manually assigned supergroup.		tax_class	-	**Yilmaz et al., 2011; Balvočiūtė and Huson, 2017; Beiko et al., 2015**
**97**	Taxonomic Annotation	manual_phylum_division	manual phylum/division	Manually assigned phylum or division.		tax_class	-	**Yilmaz et al., 2011; Balvočiūtė and Huson, 2017; Beiko et al., 2015**
**98**	Taxonomic Annotation	manual_class	manual class	Manually assigned class.		tax_class	-	**Yilmaz et al., 2011; Balvočiūtė and Huson, 2017; Beiko et al., 2015**
**99**	Taxonomic Annotation	manual_order	manual order	Manually assigned order.		tax_class	-	**Yilmaz et al., 2011; Balvočiūtė and Huson, 2017; Beiko et al., 2015**
**100**	Taxonomic Annotation	manual_family	manual family	Manually assigned family.		tax_class	-	**Yilmaz et al., 2011; Balvočiūtė and Huson, 2017; Beiko et al., 2015**
**101**	Taxonomic Annotation	manual_genus	manual genus	Manually assigned genus.		tax_class	-	**Yilmaz et al., 2011; Balvočiūtė and Huson, 2017; Beiko et al., 2015**
**102**	Taxonomic Annotation	manual_species	manual species	Manually assigned species.		tax_class	-	**Yilmaz et al., 2011; Balvočiūtė and Huson, 2017; Beiko et al., 2015**
**103**	Taxonomic Annotation	manual_notes	manual notes	Manual annotation notes.				

Supplementary Table S6: Freezer inventory module fields: Expanded description of all fields (field, name), associated tables (table), field examples (example), related MIxS fields (MIxS), MIMARKS-SURVEY requirements (M-S), and field citations (citations) for the Freezer Inventory module.

id	table	field	name	description	example	MIxS	M-S	citations
**1**	Return Action	action_code	action code	The code of the return action.	[none, run_result, run_prep, final_pooled_lib, pooled_lib, lib_prep, qpcr, ddpcr, extraction]			
**2**	Return Action	action_label	action label	The label of the return action.	[none, run result, run prep, final pooled library, pooled library, library prep, qPCR, ddPCR, extraction]			
**3**	Return Action	created_datetime	created datetime	The date and time the return action was created.				
**4**	Return Action	created_by	created by	The username that created the return action. This is a foreign key to the Users table.				
**5**	Freezer	freezer_label	freezer label	The label of the freezer.	freezer 1			
**6**	Freezer	freezer_room_name	freezer room name	The name of the room the freezer is located in.	Murray 313			
**7**	Freezer	freezer_depth	freezer depth	The depth of the freezer.				
**8**	Freezer	freezer_length	freezer length	The length of the freezer.				
**9**	Freezer	freezer_width	freezer width	The width of the freezer.				
**10**	Freezer	freezer_dimension_units	freezer dimension units	The units of the depth, length, and width. This references the enumeration MeasureUnits.	[meter (m), centimeter (cm), feet (ft), inches (in)]			
**11**	Freezer	freezer_capacity_columns	freezer capacity columns	The maximum number of columns that can fit in the freezer, where the unit is a freezer box.	10			
**12**	Freezer	freezer_capacity_rows	freezer capacity rows	The maximum number of rows that can fit in the freezer, where the unit is a freezer box.	10			
**13**	Freezer	freezer_capacity_depth	freezer capacity depth	The maximum depth that can fit in the freezer, where the unit is a freezer box.	10			
**14**	Freezer	freezer_rated_temp	freezer_rated_temp	temperature at which sample was stored	-80	samp_store_temp	X	**Bustin et al., 2009; Yilmaz et al., 2011**

id	table	field	name	description	example	MIxS	M-S	citations
15	Freezer	freezer_rated_temp_units	freezer_rated_temp_units	temperature units at which sample was stored. This references the enumeration TempUnits.	[Celsius, Fahrenheit, Kelvin]	samp_store_temp	X	**Bustin et al., 2009; Yilmaz et al., 2011**
16	Freezer	created_datetime	created datetime	The date and time the freezer was created.				
17	Freezer	created_by	created by	The username that created the freezer. This is a foreign key to the Users table.				
18	Freezer Rack	freezer	freezer	The associated freezer of the freezer rack. This is a foreign key to the freezer table.		samp_store_loc	X	**Bustin et al., 2009; Yilmaz et al., 2011**
19	Freezer Rack	freezer_rack_label	freezer rack label	The label of the freezer rack.	freezer rack 1			
20	Freezer Rack	freezer_rack_column_start	freezer rack column start	The start column location of the freezer rack in the freezer. Grid units are in boxes specified in freezer_capacity fields.				
21	Freezer Rack	freezer_rack_column_end	freezer rack column end	The end column location of the freezer rack in the freezer. Grid units are in boxes specified in freezer_capacity fields.				
22	Freezer Rack	freezer_rack_row_start	freezer rack row start	The start row location of the freezer rack in the freezer. Grid units are in boxes specified in freezer_capacity fields.				
23	Freezer Rack	freezer_rack_row_end	freezer rack row end	The end row location of the freezer rack in the freezer. Grid units are in boxes specified in freezer_capacity fields.				
24	Freezer Rack	freezer_rack_depth_start	freezer depth start	The start depth of the freezer rack in the freezer. Grid units are in boxes specified in freezer_capacity fields.				

id	table	field	name	description	example	MIxS	M-S	citations
**25**	Freezer Rack	freezer_rack_depth_end	freezer depth end	The end depth of the freezer rack in the freezer. Grid units are in boxes specified in freezer_capacity fields.				
**26**	Freezer Rack	created_datetime	created datetime	The date and time the freezer was created.				
**27**	Freezer Rack	created_by	created by	The username that created the freezer rack. This is a foreign key to the Users table.				
**28**	Freezer Box	freezer_rack	freezer rack	The associated freezer rack of the freezer box. This is a foreign key to the Freezer Rack table.		samp_store_loc	X	**Bustin et al., 2009; Yilmaz et al., 2011**
**29**	Freezer Box	freezer_box_label	freezer box label	The label of the freezer box.	box 1			
**30**	Freezer Box	freezer_box_column	freezer box column	The column location of the freezer box in the freezer rack. Grid units are in boxes specified in freezer_capacity fields.				
**31**	Freezer Box	freezer_box_row	freezer box row	The row location of the freezer box in the freezer rack. Grid units are in boxes specified in freezer_capacity fields.				
**32**	Freezer Box	freezer_box_depth	freezer box depth	The depth location of the freezer box in the freezer rack. Grid units are in boxes specified in freezer_capacity fields.				
**33**	Freezer Box	freezer_box_capacity_column	freezer box capacity column	The maximum number of columns that can fit in the freezer box, where the unit is sample inventory.				
**34**	Freezer Box	freezer_box_capacity_row	freezer box capacity row	The maximum number of rows that can fit in the freezer box, where the unit is sample inventory.				
**35**	Freezer Box	created_datetime	created datetime	The date and time the freezer box was created.				

id	table	field	name	description	example	MIxS	M-S	citations
**36**	Freezer Box	created_by	created by	The username that created the freezer box. This is a foreign key to the Users table.				
**37**	Freezer Inventory	freezer_box	freezer box	The associated freezer box of the freezer inventory. This is a foreign key to the Freezer Box table.		samp_store_loc	X	**Bustin et al., 2009; Yilmaz et al., 2011**
**38**	Freezer Inventory	sample_barcode	sample barcode	The associated sample barcode of the freezer inventory. This is a foreign key to the Sample Barcode table.				
**39**	Freezer Inventory	freezer_inventory_type	freezer inventory type	The freezer inventory type. This references the enumeration InvTypes.	[filter, subcore, extraction, pooled library]			
**40**	Freezer Inventory	freezer_inventory_status	freezer inventory status	The freezer inventory status. This references the enumeration InvStatus.	[in stock, checked out, permanently removed]			
**41**	Freezer Inventory	freezer_inventory_column	freezer inventory column	The column location of the freezer inventory in the freezer box. Grid units are in inventory specified in freezer_box_capacity fields.				
**42**	Freezer Inventory	freezer_inventory_row	freezer inventory row	The row location of the freezer inventory in the freezer box. Grid units are in inventory specified in freezer_box_capacity fields.				
**43**	Freezer Inventory	freezer_inventory_freeze_datetime	freezer inventory freeze datetime	The date and time the freezer inventory was frozen for the first time.		samp_store_dur	X	**Bustin et al., 2009; Yilmaz et al., 2011**
**44**	Freezer Inventory	created_datetime	created datetime	The date and time the freezer inventory was created.		samp_store_dur	X	**Bustin et al., 2009; Yilmaz et al., 2011**
**45**	Freezer Inventory	created_by	created by	The username that created the freezer inventory. This is a foreign key to the Users table.				

id	table	field	name	description	example	MIxS	M-S	citations
46	Freezer Inventory Log	freezer_inventory	freezer inventory	The associated freezer inventory of the freezer log. This is a foreign key to the Freezer Inventory table.				
47	Freezer Inventory Log	freezer_log_action	inventory log action	The action of the freezer inventory log. This references the enumeration InvStatus.	[checkout, return, permanent removal]			
48	Freezer Inventory Log	freezer_log_notes	inventory log notes	The notes of the freezer inventory log.				
49	Freezer Inventory Log	created_datetime	created datetime	The date and time the freezer inventory log was created.				
50	Freezer Inventory Log	created_by	created by	The username that created the freezer inventory log. This is a foreign key to the Users table.				
51	Freezer Inventory Return Metadata	freezer_log	freezer log	The associated freezer inventory log. This is a foreign key to the Freezer Inventory Log table.				
52	Freezer Inventory Return Metadata	freezer_return_metadata_entered	metadata entered	Boolean indication of whether inventory return metadata was entered. This references the enumeration YesNo.	[yes, no]			
53	Freezer Inventory Return Metadata	freezer_return_actions	return actions	Each associated return action. This is a foreign key to the Return Action table.	[none, run_result, run_prep, final_pooled_lib, pooled_lib, lib_prep, qpcr, ddpcr, extraction]			
54	Freezer Inventory Return Metadata	freezer_return_vol_taken	return volume taken	If volume was taken from the sample, the amount withdrawn.				
55	Freezer Inventory Return Metadata	freezer_return_vol_units	return volume units	If volume was taken from the sample, the units of the amount withdrawn. This references the enumeration VolUnits.	[microliter (µL), milliliter (mL)]			
56	Freezer Inventory Return Metadata	freezer_return_notes	return notes	The notes of the return.				
57	Freezer Inventory Return Metadata	created_datetime	created datetime	The date and time the return was created.				
58	Freezer Inventory Return Metadata	created_by	created by	The username that created the return. This is a foreign key to the Users table.				

Supplementary Table S7: Supplementary table references: List of all citations used in Supplementary Tables, including each reference title, relevance, citation, DOI, and full citation in APA format.

id	title	relevance	citation	doi	bibliography
1	Basic local alignment search tool	Annotation method (BLAST)	Altschul et al., 1990	https://doi.org/10.1016/S0022-2836(05)80360-2	**Altschul, S. F. et al. (1990). Basic local alignment search tool. Journal of Molecular Biology, 215(3), 403–410. https://doi.org/10.1016/S0022-2836(05)80360-2**
2	Deblur Rapidly Resolves Single-Nucleotide Community Sequence Patterns	Denoise method	Amir et al., 2017	https://doi.org/10.1128/mSystems.00191-16	**Amir, A. et al. (2017). Deblur Rapidly Resolves Single-Nucleotide Community Sequence Patterns. MSystems, 2(2). https://doi.org/10.1128/mSystems.00191-16**
3	SILVA, RDP, Greengenes, NCBI and OTT—how do these taxonomies compare?	Reference database	Balvočiūtė and Huson, 2017	https://doi.org/10.1186/s12864-017-3501-4	**Balvočiūtė, M., & Huson, D. H. (2017). SILVA, RDP, Greengenes, NCBI and OTT — how do these taxonomies compare? BMC Genomics, 18(S2), 114. https://doi.org/10.1186/s12864-017-3501-4**
4	Environmental Conditions Influence eDNA Persistence in Aquatic Systems	Temperature, salinity, dissolved oxygen, ultraviolet radiation, sediment, salinity, and pH affect DNA degredation	Barnes et al., 2014	https://doi.org/10.1021/es404734p	**Barnes, M. A. et al. (2014). Environmental Conditions Influence eDNA Persistence in Aquatic Systems. Environmental Science & Technology, 48(3), 1819–1827. https://doi.org/10.1021/es404734p**
5	The ecology of environmental DNA and implications for conservation genetics	field collection; water sample collection; water temperature	Barnes and Turner, 2016	https://doi.org/10.1007/s10592-015-0775-4	**Barnes, M. A., & Turner, C. R. (2016). The ecology of environmental DNA and implications for conservation genetics. Conservation Genetics, 17(1), 1–17. https://doi.org/10.1007/s10592-015-0775-4**
6	Microbial Malaise: How Can We Classify the Microbiome?	Taxonomy assignment	Beiko, 2015	https://doi.org/10.1016/j.tim.2015.08.009	**Beiko, R. G. (2015). Microbial Malaise: How Can We Classify the Microbiome? Trends in Microbiology, 23(11), 671–679. https://doi.org/10.1016/j.tim.2015.08.009**
7	Optimizing taxonomic classification of marker-gene amplicon sequences with QIIME 2's q2-feature-classifier plugin	Annotation method (q2-feature-classifier)	Bokulich et al., 2018	https://doi.org/10.1186/s40168-018-0470-z	**Bokulich, N. A. et al. (2018). Optimizing taxonomic classification of marker-gene amplicon sequences with QIIME 2's q2-feature-classifier plugin. Microbiome, 6(1), 90. https://doi.org/10.1186/s40168-018-0470-z**
8	Reproducible, interactive, scalable and extensible microbiome data science using QIIME 2	Software package (bioinformatics)	Bolyen et al., 2019	https://doi.org/10.1038/s41587-019-0209-9	**Bolyen, E. et al. (2019). Reproducible, interactive, scalable and extensible microbiome data science using QIIME 2. Nature Biotechnology, 37(8), 852–857. https://doi.org/10.1038/s41587-019-0209-9**
9	Improved protocols for Illumina sequencing	Wet lab: library prep: size selection	Bronner et al., 2014	https://doi.org/10.1002/0471142905.hg1802s79	**Bronner, I. F. et al. (2014). Improved Protocols for Illumina Sequencing. Current Protocols in Human Genetics, 80, 18.2.1. https://doi.org/10.1002/0471142905.hg1802s79**

id	title	relevance	citation	doi	bibliography
10	Toward a global reference database of COI barcodes for marine zooplankton	Reference database (COI - MetaZooGene Barcode Atlas and Database (MZGdb))	Bucklin et al., 2021	https://doi.org/10.1007/s00227-021-03887-y	**Bucklin, A. et al. (2021). Toward a global reference database of COI barcodes for marine zooplankton. Marine Biology, 168(6), 78. https://doi.org/10.1007/s00227-021-03887-y**
11	The MIQE Guidelines: Minimum Information for Publication of Quantitative Real-Time PCR Experiments. Clinical Chemistry	Minimum standards for submission of PCR results to open access databases.	Bustin et al., 2009	https://doi.org/10.1373/clinchem.2008.112797	**Bustin, S. A. et al. (2009). The MIQE Guidelines: Minimum Information for Publication of Quantitative Real-Time PCR Experiments. Clinical Chemistry, 55(4), 611–622. https://doi.org/10.1373/clinchem.2008.112797**
12	Does size matter? An experimental evaluation of the relative abundance and decay rates of aquatic eDNA	Field collection and wet lab information; sampling protocols, primer information, extraction protocols	Bylemans et al., 2018	https://doi.org/10.1021/acs.est.8b01071	**Bylemans, J. et al. (2018). Does Size Matter? An Experimental Evaluation of the Relative Abundance and Decay Rates of Aquatic Environmental DNA. Environmental Science & Technology, 52(11), 6408–6416. https://doi.org/10.1021/acs.est.8b01071**
13	DADA2: High-resolution sample inference from Illumina amplicon data	Denoise method	Callahan et al., 2016	https://doi.org/10.1038/nmeth.3869	**Callahan, B. J. et al. (2016). DADA2: High-resolution sample inference from Illumina amplicon data. Nature Methods, 13(7), 581–583. https://doi.org/10.1038/nmeth.3869**
14	BLAST+: architecture and applications	Annotation method (BLAST+)	Camacho et al., 2009	https://doi.org/10.1186/1471-2105-10-421	**Camacho, C. et al. (2009). BLAST+: Architecture and applications. BMC Bioinformatics, 10(1), 421. https://doi.org/10.1186/1471-2105-10-421**
15	Non-specific amplification compromises environmental DNA metabarcoding with COI	Wet lab info; primers: target location, gene, length	Collins et al., 2019	https://doi.org/10.1111/2041-210X.13276	**Collins, R. A. et al. (2019). Non-specific amplification compromises environmental DNA metabarcoding with COI. Methods in Ecology and Evolution, 10(11), 1985–2001. https://doi.org/10.1111/2041-210X.13276**
16	The minimum information about a genome sequence (MIGS) specification	Minimum standards for submission of sequences to open access databases.	Field et al., 2008	https://doi.org/10.1038/nbt1360	**Field, D. et al. (2008). The minimum information about a genome sequence (MIGS) specification. Nature Biotechnology, 26(5), 541–547. https://doi.org/10.1038/nbt1360**
17	The importance of molecular markers and primer design when characterizing biodiversity from environmental DNA	primer design; wet lab	Freeland, 2017	https://doi.org/10.1139/gen-2016-0100	**Freeland, J. R. (2017). The importance of molecular markers and primer design when characterizing biodiversity from environmental DNA. Genome, 60(4), 358–374. https://doi.org/10.1139/gen-2016-0100**
18	The Protist Ribosomal Reference database (PR2): a catalog of unicellular eukaryote Small Sub-Unit rRNA sequences with curated taxonomy	Reference database	Guillou et al., 2012	https://doi.org/10.1093/nar/gks1160	**Guillou, L. et al. (2012). The Protist Ribosomal Reference database (PR2): A catalog of unicellular eukaryote Small Sub-Unit rRNA sequences with curated taxonomy. Nucleic Acids Research, 41(D1), D597–D604. https://doi.org/10.1093/nar/gks1160**

id	title	relevance	citation	doi	bibliography
19	Predicting the fate of eDNA in the environment and implications for studying biodiversity	Field collection standardization, ecosystem specific parameterization of eDNA, recommendation for metadata collection field collection	Harrison et al., 2019	https://doi.org/10.1098/rspb.2019.1409	**Harrison, J. B. et al. (2019). Predicting the fate of eDNA in the environment and implications for studying biodiversity. Proceedings of the Royal Society B: Biological Sciences, 286(1915), 20191409. https://doi.org/10.1098/rspb.2019.1409**
20	MitoFish and MitoAnnotator: A Mitochondrial Genome Database of Fish with an Accurate and Automatic Annotation Pipeline	Reference database (12S - MitoFish)	Iwasaki et al., 2013	https://doi.org/10.1093/molbev/mst141	**Iwasaki, W. et al. (2013). MitoFish and MitoAnnotator: A Mitochondrial Genome Database of Fish with an Accurate and Automatic Annotation Pipeline. Molecular Biology and Evolution, 30(11), 2531–2540. https://doi.org/10.1093/molbev/mst141**
21	Estimating fish abundance and biomass from eDNA concentrations: variability among capture methods and environmental conditions	Field collection; filtration method, filter selection	Lacoursière-Roussel et al., 2016	https://doi.org/10.1111/1755-0998.12522	**Lacoursière-Roussel, A. et al. (2016). Estimating fish abundance and biomass from eDNA concentrations: Variability among capture methods and environmental conditions. Molecular Ecology Resources, 16(6), 1401–1414. https://doi.org/10.1111/1755-0998.12522**
22	An illustrated manual for environmental DNA research: Water sampling guidelines and experimental protocols	Field collection and wet lab information; field protocols, extraction protocols	Minamoto et al., 2021	https://doi.org/10.1002/edn3.121	**Minamoto, T. et al. (2021). An illustrated manual for environmental DNA research: Water sampling guidelines and experimental protocols. Environmental DNA, 3(1), 8–13. https://doi.org/10.1002/edn3.121**
23	MiFish, a set of universal PCR primers for metabarcoding environmental DNA from fishes: detection of more than 230 subtropical marine species	Wet lab information (primers, metabarcoding)	Miya et al., 2015	https://doi.org/10.1098/rsos.150088	**Miya, M. et al. (2015). MiFish, a set of universal PCR primers for metabarcoding environmental DNA from fishes: Detection of more than 230 subtropical marine species. Royal Society Open Science, 2(7), 150088. https://doi.org/10.1098/rsos.150088**
24	An analysis of metadata reporting in freshwater environmental DNA research calls for the development of best practice guidelines	Metadata reporting for eDNA; Field collection: sample collection, type; Wet lab: extraction, filtration,	Nicholson et al., 2020	https://doi.org/10.1002/edn3.81	**Nicholson, A. et al. (2020). An analysis of metadata reporting in freshwater environmental DNA research calls for the development of best practice guidelines. Environmental DNA, 2(3), 343–349. https://doi.org/10.1002/edn3.81**
25	Environmental DNA metabarcoding for benthic monitoring: A review of sediment sampling and DNA extraction methods	Field collection and wet lab information; sediment sampling protocols, extraction methods	Pawlowski et al., 2022	https://doi.org/10.1016/j.scitotenv.2021.151783	**Pawlowski, J. et al. (2022). Environmental DNA metabarcoding for benthic monitoring: A review of sediment sampling and DNA extraction methods. Science of The Total Environment, 818, 151783. https://doi.org/10.1016/j.scitotenv.2021.151783**
26	The detection of aquatic animal species using enviornmental DNA- a review of eDNA as a survey tool in ecology	Field collection and wet lab info; water sample collection, primer validation	Rees et al., 2014	https://doi.org/10.1111/1365-2664.12306	**Rees, H. C. et al. (2014). REVIEW: The detection of aquatic animal species using environmental DNA - a review of eDNA as a survey tool in ecology. Journal of Applied Ecology, 51(5), 1450–1459. https://doi.org/10.1111/1365-2664.12306**

id	title	relevance	citation	doi	bibliography
**27**	MitoFish and MiFish Pipeline: A Mitochondrial Genome Database of Fish with an Analysis Pipeline for Environmental DNA Metabarcoding	Reference database (12S - MitoFish)	Sato et al., 2018	https://doi.org/10.1093/molbev/msy074	**Sato, Y. et al. (2018). MitoFish and MiFish Pipeline: A Mitochondrial Genome Database of Fish with an Analysis Pipeline for Environmental DNA Metabarcoding. Molecular Biology and Evolution, 35(6), 1553–1555. https://doi.org/10.1093/molbev/msy074**
**28**	Introducing mothur: Open-Source, Platform-Independent, Community-Supported Software for Describing and Comparing Microbial Communities	Software package (bioinformatics)	Schloss et al., 2009	https://doi.org/10.1128/AEM.01541-09	**Schloss, P. D. et al. (2009). Introducing mothur: Open-Source, Platform-Independent, Community-Supported Software for Describing and Comparing Microbial Communities. Applied and Environmental Microbiology, 75(23), 7537–7541. https://doi.org/10.1128/AEM.01541-09**
**29**	Quantifying effects of UV-B, temperature, and pH on eDNA degradation in aquatic microcosms.	field collection; water sample collection	Strickler et al., 2015	https://doi.org/10.1016/j.biocon.2014.11.038	**Strickler, K. M. et al. (2015). Quantifying effects of UV-B, temperature, and pH on eDNA degradation in aquatic microcosms. Biological Conservation, 183, 85–92. https://doi.org/10.1016/j.biocon.2014.11.038**
**30**	On-site filtration of water samples for environmental DNA analysis to avoid DNA degradation during transportation	Temperature and time to filtration affect DNA degredation	Yamanaka et al., 2016	https://doi.org/10.1007/s11284-016-1400-9	**Yamanaka, H. et al. (2016). On-site filtration of water samples for environmental DNA analysis to avoid DNA degradation during transportation. Ecological Research, 31(6), 963–967. https://doi.org/10.1007/s11284-016-1400-9**
**31**	Minimum information about a marker gene sequence (MIMARKS) and minimum information about any (x) sequence (MIxS) specifications	Minimum standards and specialty specifications for submission of sequences to open access databases.	Yilmaz et al., 2011	https://doi.org/10.1038/nbt.1823	**Yilmaz, P. et al. (2011). Minimum information about a marker gene sequence (MIMARKS) and minimum information about any (x) sequence (MIxS) specifications. Nature Biotechnology, 29(5), 415–420. https://doi.org/10.1038/nbt.1823**
**32**	The SILVA and “All-species Living Tree Project (LTP)” taxonomic frameworks	Reference database	Yilmaz et al., 2014	https://doi.org/10.1093/nar/gkt1209	**Yilmaz, P. et al. (2014). The SILVA and “All-species Living Tree Project (LTP)” taxonomic frameworks. Nucleic Acids Research, 42(D1), D643–D648. https://doi.org/10.1093/nar/gkt1209**
